# Stromal microenvironment promoted infiltration in esophageal adenocarcinoma and squamous cell carcinoma: a multi-cohort gene-based analysis

**DOI:** 10.1038/s41598-020-75541-4

**Published:** 2020-10-29

**Authors:** Jiali Li, Zihang Zeng, Xueping Jiang, Nannan Zhang, Yanping Gao, Yuan Luo, Wenjie Sun, Shuying Li, Jiangbo Ren, Yan Gong, Conghua Xie

**Affiliations:** 1 Department of Radiation and Medical Oncology, Zhongnan Hospital of Wuhan University, Wuhan, China; 2 Department of Biological Repositories, Zhongnan Hospital of Wuhan University, Wuhan, China; 3 Human Genetics Resource Preservation Center of Hubei Province, Zhongnan Hospital of Wuhan University, Wuhan, China; 4 Hubei Key Laboratory of Tumor Biological Behaviors, Zhongnan Hospital of Wuhan University, Wuhan, China; 5 Hubei Cancer Clinical Study Center, Zhongnan Hospital of Wuhan University, Wuhan, China

**Keywords:** Tumour biomarkers, Bioinformatics, Biological models, Cancer microenvironment, Computational biology and bioinformatics

## Abstract

The stromal microenvironment has been shown to affect the infiltration of esophageal carcinoma (ESCA), which is linked to prognosis. However, the complicated mechanism of how infiltration is influenced by the stromal microenvironment is not well-defined. In this study, a stromal activation classifier was established with ridge cox regression to calculate stroma scores for training (n = 182) and validation cohorts (n = 227) based on the stroma-related 32 hub genes identified by sequential bioinformatics algorithms. Patients with high stromal activation were associated with high T stage and poor prognosis in both esophagus adenocarcinoma and esophagus squamous cell carcinoma. Besides, comprehensive multi-omics analysis was used to outline stromal characterizations of 2 distinct stromal groups. Patients with activated tumor stoma showed high stromal cell infiltration (fibroblasts, endothelial cells, and monocyte macrophages), epithelial-mesenchymal transition, tumor angiogenesis and M2 macrophage polarization (CD163 and CD206). Tumor mutation burden of differential stromal groups was also depicted. In addition, a total of 6 stromal activation markers in ESCA were defined and involved in the function of carcinoma-associated fibroblasts that were crucial in the differentiation of distinct stromal characterizations. Based on these studies, a practical classifier for the stromal microenvironment was successfully proposed to predict the prognosis of ESCA patients.

## Introduction

Esophageal carcinoma (ESCA), consisting of esophagus adenocarcinoma (ESAD) and esophagus squamous cell carcinoma (ESCC), is one of the most frequently occurring gastrointestinal cancers, causing about 375,000 deaths annually worldwide^1^. The morbidity and mortality of ESCA remain high in most area even though different therapeutic treatments are provided. Around 75% patients were at advanced stages when first diagnosed. Excessive infiltration and distant metastasis lead to the failure of surgery and those patients will end up with a terminal and fetal carcinoma status^2^. Moreover, advanced tumor is comparatively tolerant to neoadjuvant chemotherapy and radiotherapy due to accumulating drug and ray resistance. The 5-years survival of advanced ESCA patients is merely 15–20%^3^.

ESCA is actually a complex ecosystem, composing cellular components as well as many other non-cell factors. An activated tumor stroma, containing over-functional fibroblasts, osteoblasts, chondrocytes, mesenchymal stromal cells and the extracellular matrix (ECM), plays a crucial role in tumor initiation, progression and metastasis. As was reported, carcinoma-associated fibroblasts (CAFs) functioned as relatively central part in tumor stroma^4^. CAFs predominated the cell mobility among ECM with a dynamic balance of secreted matrix metalloproteinases (MMPs) and fibrous macromolecules^5^. CAF-derived transform growth factor-β (TGF-β) modified the process of epithelial-mesenchymal transition (EMT) during tumor infiltration in breast cancer^6^. Similarly, CAFs were demonstrated as the major sources of chemokines, releasing CXCL12 to recruit monocytes as tumor-associated macrophages (TAMs) in tumor microenvironment^7^. In addition, CAFs promoted tumor angiogenesis in tumor stroma with vascular endothelial growth factor (VEGF) secretion to reconstruct blood vessel^8^. It was reported that the stroma facilitated the acquisition of stem-like properties of cancer cells, rendering oncocytes ability to colonize and infiltration^9,10^. Tumor stroma had lots of crosstalks with tumor cells and could shape into tumor- suppressing or tumor-promoting microenvironment^5^. However, an evaluation index for stroma activation and the impact of stroma activation on patient survival remains unclear, especially at the population level.

**Figure 1:**
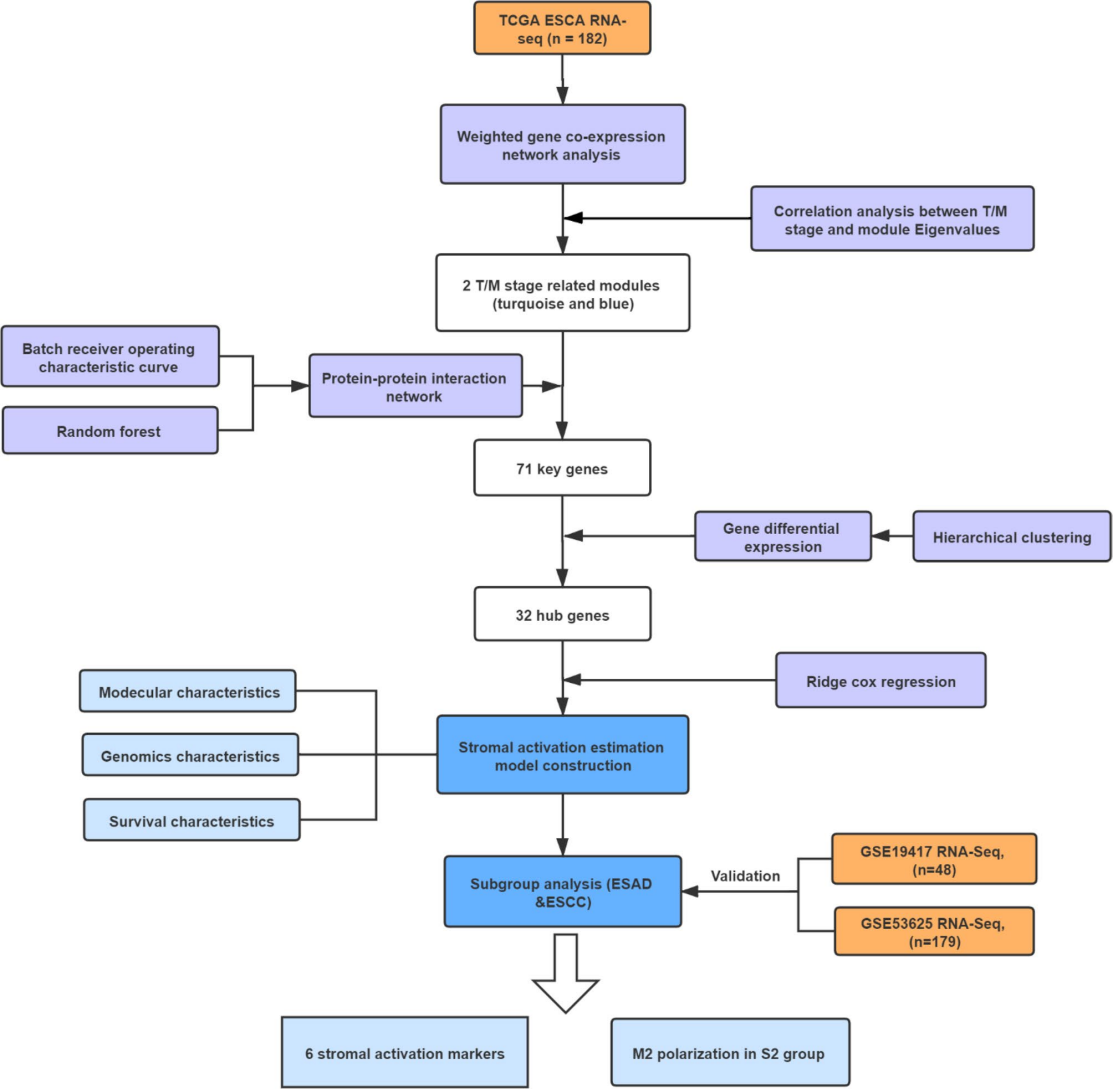
Workflow of this study.

Accumulating researches have indicated the essential roles of stroma in stroma-tumor complex and potential importance in the survival of tumor patients^11,12^. In this study, we identified featured genes that were correlated with ESCA infiltration and stromal elements in the training (n = 182) and validation cohorts (n = 227). A stromal activation estimation model was established based on the stroma-related hub genes by ridge cox regression. ESCA patients were divided into 2 subtypes with different stromal activation indexes. Patients in high stromal activation had more stromal cell infiltration, EMT and TAM polarization, as well as tumor angiogenesis, all of which contributed to worse survival. The crucial roles of CAFs in tumor stroma were highlighted with the identification of 6 stroma markers (MMP11, COL6A2, COL1A2, CTHRC1, FAP, and LUM). Our study proposed a novel model to evaluate stromal status and descripted 2 distinct molecular, cellular, and clinical characteristics in ESCA patients. The workflow of this study was provided in Fig. 1.

## Results

### Infiltration was associated with stromal-related gene patterns in ESCA

**Figure 2:**
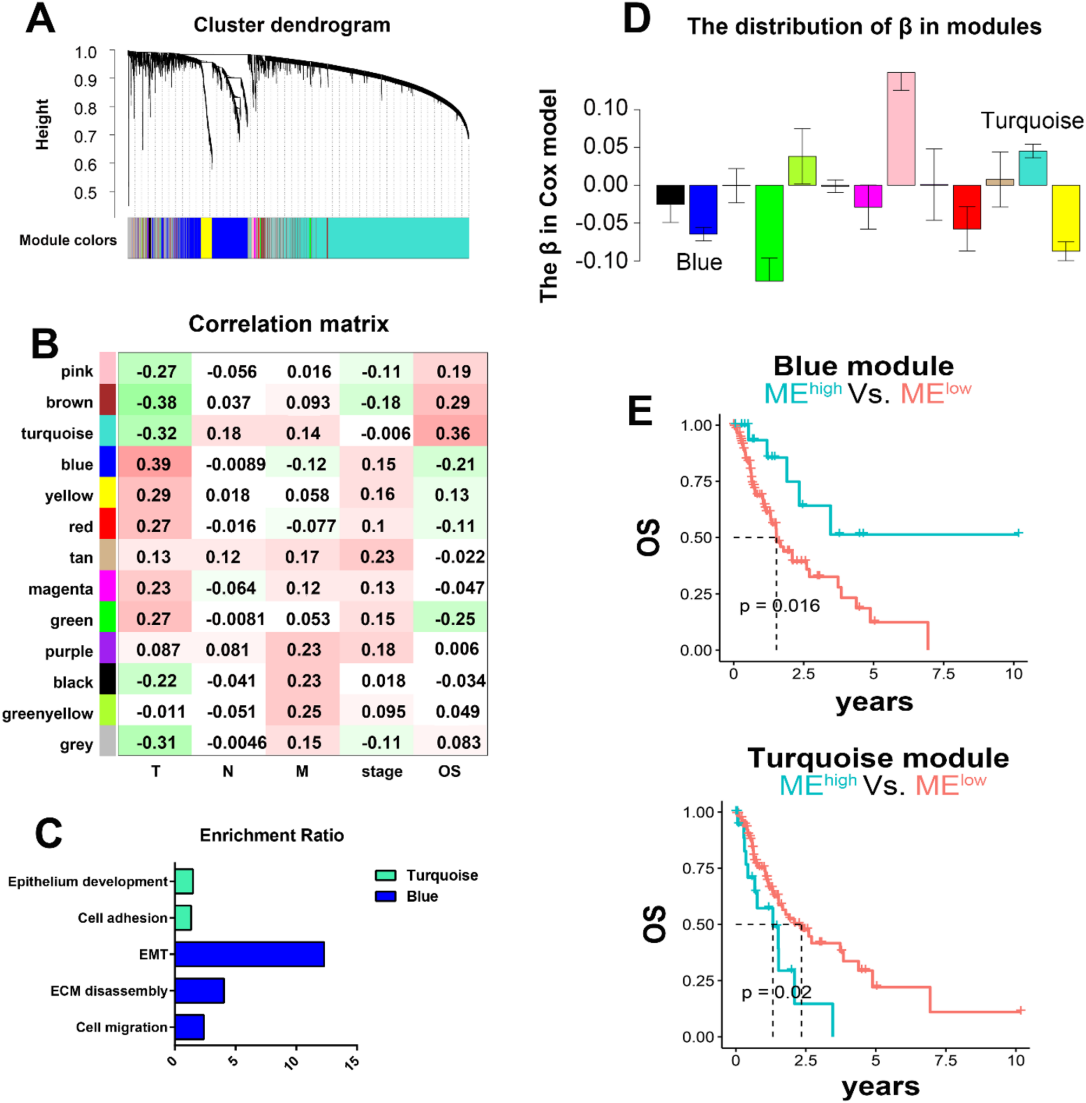
Co-expression gene modules with stromal signal in ESCA. **(A)** Categorization of genes into different modules with dendrogram clustering based on dissimilarity calculated by topological overlap. **(B)** Correlations among 13 modules and clinical traits illustrated by different colors. **(C)** Enrichment analysis of stromal-related modules. **(D)** Distribution of β in the Cox model. **(E)** Survival curve of high-low eigenvalues in stromal-related modules.

Gene expression patterns were identified in the training cohort with mRNA-Seq of ESCA patients from The Cancer Genome Atlas (TCGA) database. After removing outlier samples by hierarchical clustering, 182 ESCA patients were included in further research as the training data. WGCNA (power = 4) was performed to descript the co-expression patterns, and 13 gene modules sharing similar expression patterns were determined based on K-means clustering and dynamic tree cut (Fig. 2A) Correlation matrix between expression models and targeted clinical characteristics including T, N, M stage, pathological stage and OS revealed the implication of feature expression on clinical phenotypes (Fig. 2B). Interestingly, blue and turquoise gene patterns showed the negative correlations with T and M stages (blue: T 0.39 (7e−08), M − 0.12 (0.1); turquoise: T − 0.32 (1e−05), M 0.14 (0.05)), indicating that near infiltration and distant metastasis were potently associated with different stromal status.

To better understand the possible mechanism, we explored the specific biological functions of these 2 gene patterns through enrichment analysis. Both patterns exhibited stromal-related signals. The blue module was significantly enriched in cell migration, extracellular matrix disassembly, and EMT (Fig. 2C; Additional file 1: Table S1), implying pro-tumor stroma type. Cell adhesion and epithelium development were identified in the turquoise module (Fig. 2C; Additional file 1: Table S2), suggesting relatively normal stroma. To investigate the impact of the 2 stromal-related patterns on prognosis in patients, the relationship between OS and the eigenvalues of modules was analyzed using a univariate Cox regression model (Fig. 2D). The results demonstrated that the blue module was linked to unfavorable prognosis, whereas the turquoise module was associated with better survival (Fig. 2E), consistent with their correlation with T/M stage (Fig. 2B).

According to the above results, we identified 2 stromal-related gene modules. Blue module had more ECM and EMT properties, and was linked to stronger near infiltration (T stage) and worse prognosis; thus, it was considered as a stromal-activation module. On the other hand, the turquoise module was relevant to cell adhesion and epithelium development, and associated with depressed infiltration and favorable survival, and was denoted as the non-activation stromal module.

### Selection of stromal-related signatures determining T/M stage selection

**Figure 3:**
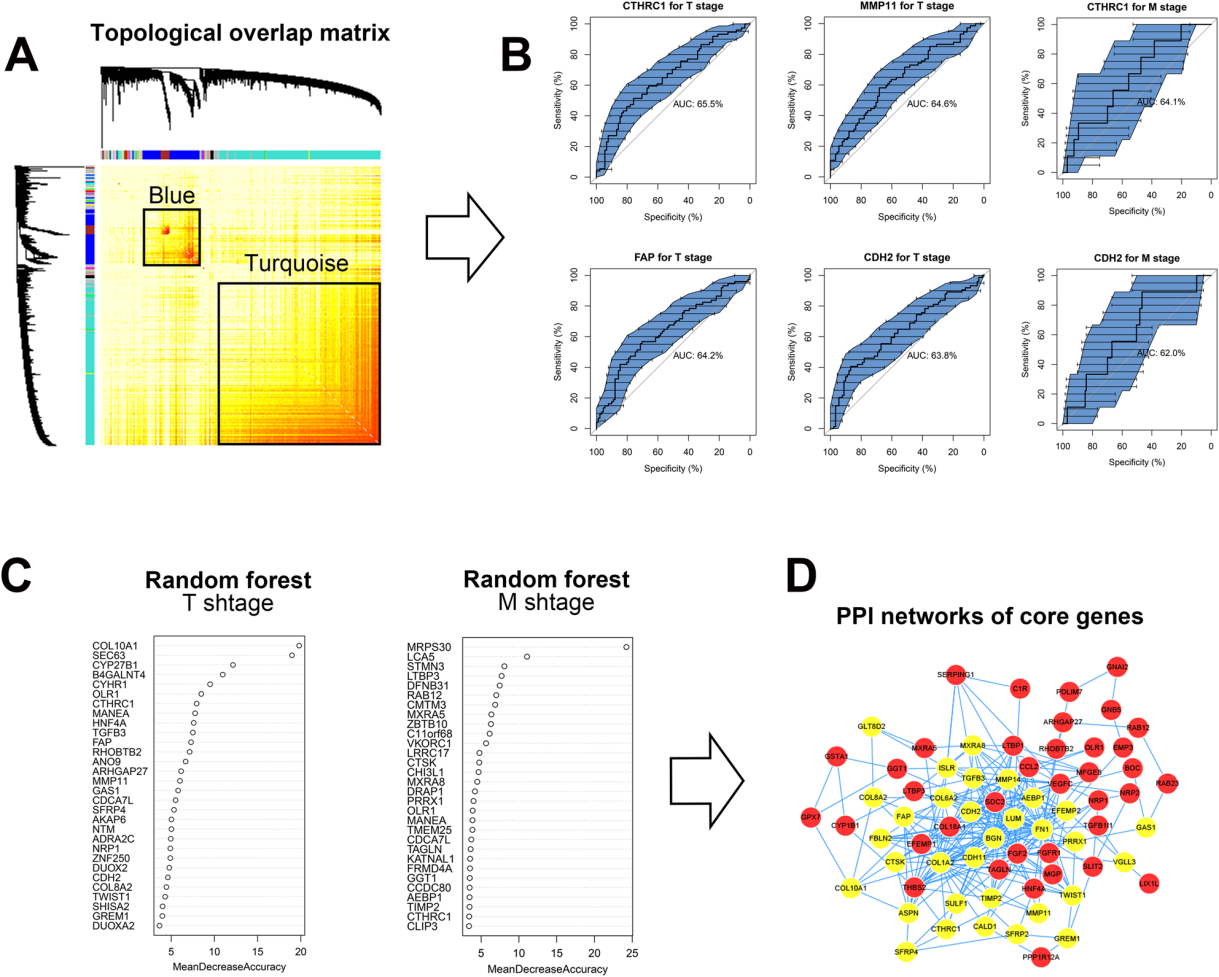
Identification of 71 hub genes through machine learning and bioinformatics in selected modules. **(A)** Genes and gene interactions by topological overlap matrix in WGCNA. The color red represents gene–gene interactions. **(B)** ROC of single gene for T/M stages. **(C)** RF of single gene for T/M stages. **(D)** Protein-protein interaction network of hub genes. WGCNA, weighted gene co-expression network analysis; RF, random forest; ROC, receiver operating characteristic curve.

To identify the stromal-related features of the 2 modules at a single-gene level (Fig. 3A), a sequential machine learning algorithm was applied. On the one hand, receiver operating characteristic (ROC) analysis was used to calculate the predictive value of single gene in blue and turquoise modules with respect to T/M stage. A total of 453 genes related to T stage and 1418 genes related to M stage (both AUC > 0.6) were identified as independent instrumental signatures in T/M stage including N-cadherin (CDH2), MMP11, fibroblast activation protein (FAP) and collagen triple helix repeat containing 1 (CTHRC1) (Fig. 3B). On the other hand, random forest (RF) algorithm was applied to estimate the contribution of each gene to T/M stage, represented by mean decrease accuracy. A total of 150 significant genes were acquired, among which TGF-β3, MMP11, and EMT markers including CDH2 and Twist Family BHLH Transcription Factor 1 (TWIST1) significantly contributed to T stage, whereas CTHRC1 and TIMP2 were the genes most associated with M stage (Fig. 3C). To extract the biological gene interaction network, the selected stromal-related signatures screened from both ROC and the RF model were submitted to protein–protein interaction (PPI) network analysis. A total of 71 genes were identified based on topological structure by PPI (higher betweenness, closeness, and degree), including collagen-related genes (COL6A2, COL8A2, COL10A1), TGFβ, MMP11, VEGF, and members of the RAS oncogene family (Fig. 3D). Gene enrichment analysis of these 71 genes (Figs. S1, S2a) indicated stromal characteristics, including cell migration, elastic fiber formation, extracellular structure organization, and vasculature development (P < 0.001, false discovery rate < 0.001, Table S3).

Hierarchical clustering was next performed to subgroup ESCA patients into 5 groups based on the expression of above 71 genes (Fig. S2b). All patients in the 5 groups exhibited stromal function according to GO enrichment results (Tables S4–S8), but could be reorganized into 2 classes with differential survival tendency. In order to obtain the dominant hub genes in prognosis, we further conducted differential gene expression (DGE) using transcriptomics data of the training cohort. The stromal-related signal was enriched in the class with worse survival, suggesting that the activation of stroma contributed to an unfavorable prognosis. As expected, the class with better survival had no stromal-related signal enrichment (Tables S9, S10). Therefore, a total of 32 highly elevated stromal signatures in worse survival class were defined as the final hub genes in ESCA stroma activation (Table S11). The 32 hub genes included MMPs family member (MMP11, MMP14), collagen-encoding genes (COL1A2, COL8A2, COL6A2, and COL10A1), EMT markers (CDH2, TWIST1), and TGF-β3.

### Stromal activation estimation model building and molecular, genomic landscape of stromal subtypes

After obtaining the 32 genes from DGE, we aimed to construct a stromal activation estimation model with these gene signatures. Stromal scores were calculated using the Cox ridge regression algorithm based on hub genes expression, by which the stromal activation of each ESCA patient was accurately reflected. Patients with higher stromal scores than the first quartile were defined as the S2 group with higher stromal activation, others as the S1 group with lower stromal activation.

**Figure 4:**
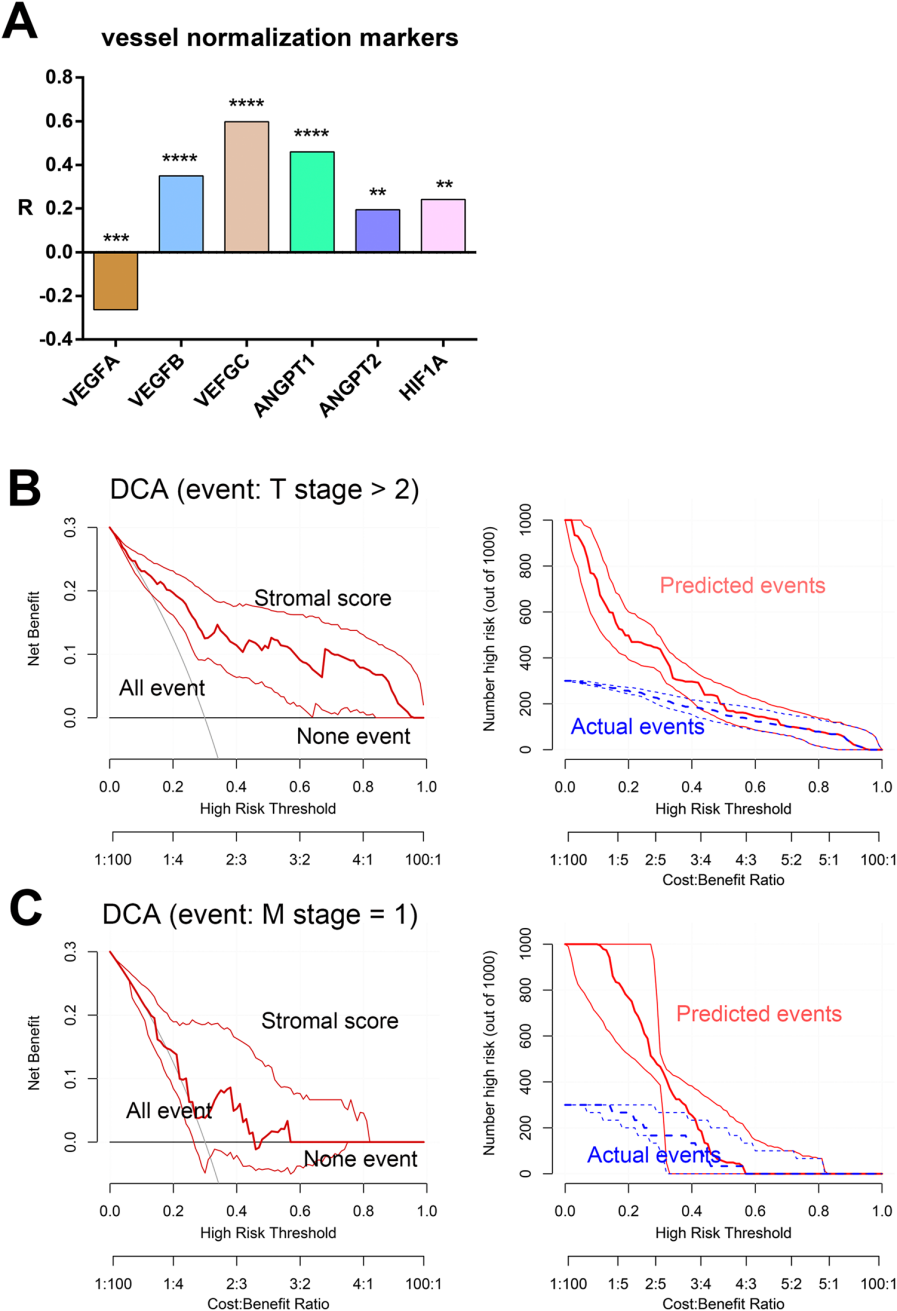
Vessel markers and DCA. **(A)** Correlations between vessel marker expression and stromal scores in the training dataset. **(B)** DCA of stromal scores for T stage prediction. **(C)** DCA of stromal scores for M stage prediction. DCA, decision curve analysis.

To confirm the efficiency of this stromal activation estimation model, we validated the relationship between the stromal scores and known markers in both training dataset. Vessel angiogenesis markers, including VEGFA, VEGFB, VEGFC, ANGPT2, and HIF1A, were correlated with high stromal scores (Fig. 4A). Next, the decision curve analysis (DCA) method was used to evaluate the prediction performance with respect to T/M stage based on stromal scores. As expected, stromal scores showed superior predictive performance for T stage compared with M stage (Fig. 4B,C), probably due to the small proportion patients with metastasis.

**Figure 5:**
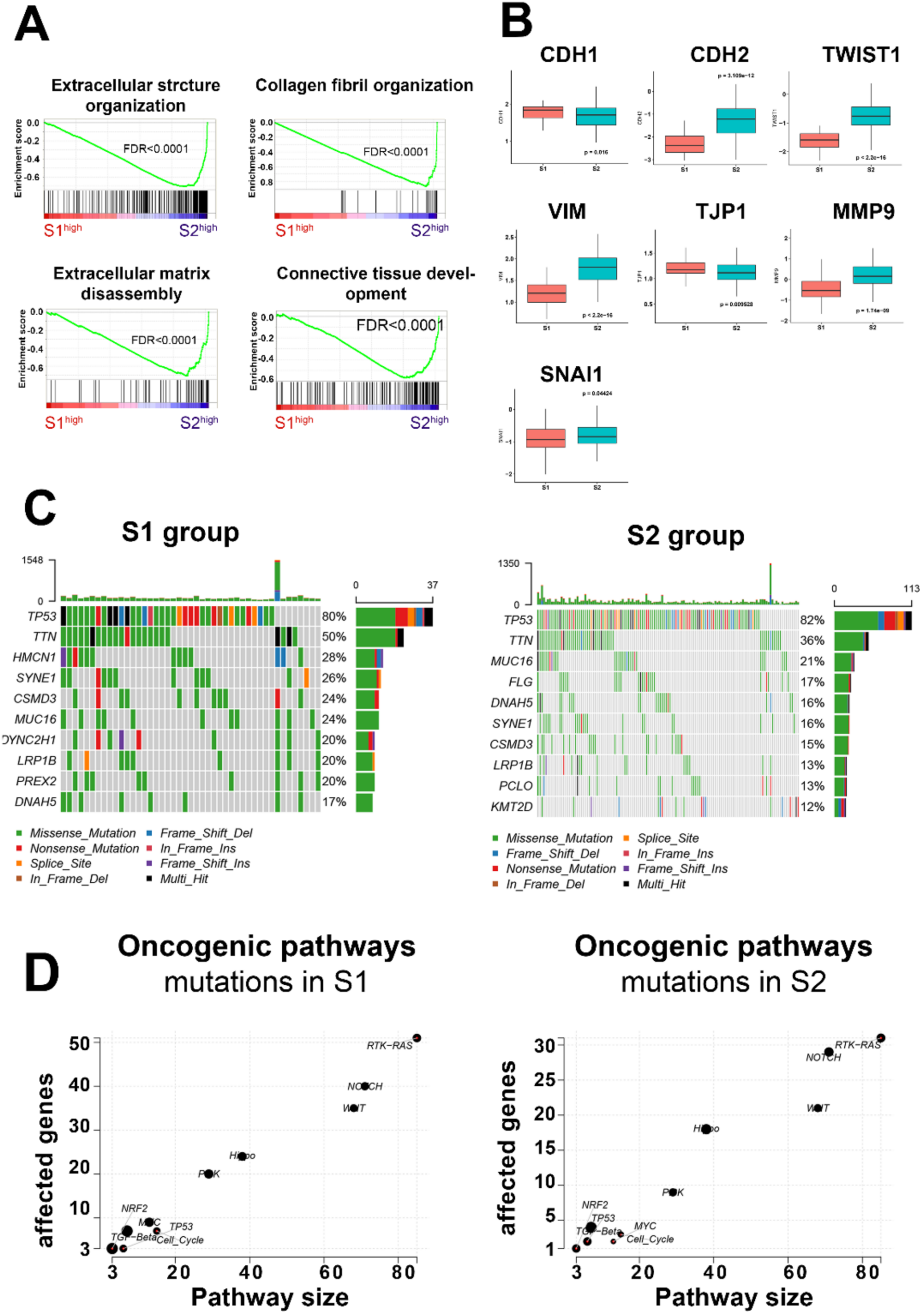
Molecular and mutation landscapes for stromal groups. **(A)** GSEA for high-low stromal score. **(B)** EMT markers in different stromal subgroups. **(C)** Top 10 mutations in the S1 and S2 groups. **(D)** Scatter plot of enrichment of known oncogenic signaling pathways in the S1 and S2 groups.

To further understand the molecular characteristics of the different subgroups of ESCA, DGE was performed for transcriptomics in the 2 stromal groups (Fig. S2c). GSEA results for DGE genes (Tables S12, S13) confirmed that collagen fibril organization, extracellular matrix disassembly, extra cellular structure organization, and connective tissue development were significantly enriched in the S2 group (Fig. 5A). In addition, the heatmap identified distinctly high expression of stromal genes (such as COLA2, FN1, and MMP14, Fig. S2d) in the S2 group. In addition, boxplot results indicated that patients in the S2 group demonstrated higher EMT activation by upregulating relevant markers such as CDH1, CDH2, vimentin (VIM), tight junction protein 1 (TJP1), snail family transcriptional repressor 1 (SNAI1), MMP9, and TWIST1 (Fig. 5B).

Next, we attempted to illustrate the heterogeneity in terms of the genome. The basic mutation information is shown in Fig. S3a. Given that tumor mutation counts were higher in the S1 group (S1:S2, 262.4 vs. 195.0, P = 0.0692, Fig. S3b) but with no significance, a further genome analysis was consequently performed to depict the portrait of the top 10 mutated signatures. TP53 gene mutation rate was similar between the 2 groups, while Titin (TTN) mutation was higher in the S1 group (Fig. 5C). Similarly, S1 patients were found to have more gene mutations in the TCGA oncogenic signaling pathways (Fig. 5D). Moreover, the S1 group tended to mutate in Tubulin Alpha 3c (TUBA3C), COL12A1, Paternally Expressed 3 (PEG3), and Microtubule-Associated Tumor Suppressor Candidate 2 (MTUS2) (Fig.S3c). These genes participate in microtubules formation, collagen synthesis and antioncogenic function relatively. On the contrary, patients in the S2 group showed higher rates of oncogenic molecule mutations, including PIK3CA and NOTCH1 mutations (Fig. S3c).

### Subgroup survival analysis and validation of the stromal subtypes in independent datasets

**Figure 6:**
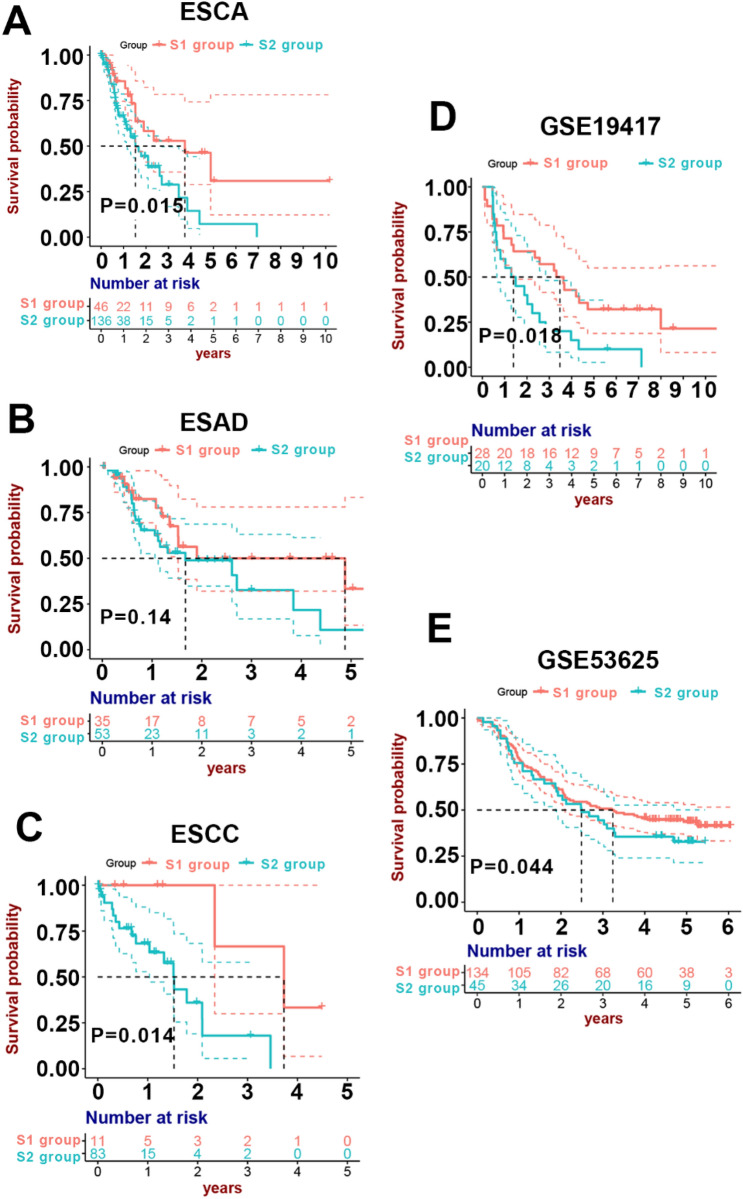
Survival analysis in training and test cohorts based on stromal group. **(A)** Survival analysis results of the TCGA ESCA dataset. **(B)** Survival analysis results of the TCGA ESAD dataset. **(C)** Survival analysis results of the TCGA ESCC dataset. **(D)** Survival analysis results of the ESAD patients in GSE19417. **(E)** Survival analysis results for the ESCC patients in GSE53625.

Survival analysis suggested that the S2 group patients with higher stromal scores had unfavorable prognosis (median survival year, S2 vs. S1: 1.53 vs. 3.73, P = 0.015, Fig. 6A), suggesting that higher stromal activation contributed to shorter survival in ESAC. Considering the biological heterogeneity between ESAD and ESCC, we performed subgroup analysis to separate these 2 types of ESCA. The S2 subgroup was more strongly associated with the T stage compared with the S1 subgroup in both ESCC and ESAD (both P-values < 0.05, Table 1). ESAD patients in the S2 group exhibited unfavorable tendency compared with low stromal activation group, although no significance was observed (P = 0.14, Fig. 6B). Notably, ESCC patients in the S2 group showed significantly worse survival due to high stromal activation (P = 0.014, Fig. 6C). All these results suggested that high stromal activation resulted in higher probability of T stage, leading to a worse prognosis.

**Table 1:** Clinical information for training patients with ESCA.

Variable	S1(ESAD)	S2(ESAD)	*P*	S1(ESCC)	S2(ESCC)	*P*
**Gender**			NS			NS
Male	31(88.6%)	45(84.9%)		8(72.7%)	72(86.7%)	
Female	4(11.4%)	8(15.1%)		3(27.3%)	11(13.3%)	
**Tobacco year (IQR)**	4.00	3.00	NS	3.00	3.00	NS
	(2.00–4.00)	(2.00–4.00)		3.00–4.50	2.00–4.00	
**Alcohol history**			NS			NS
Yes	27(79.4%)	33(62.3%)		8(72.7%)	21(25.9%)	
No	7(20.6%)	20(37.7%)		3(27.3%)	60(74.1%)	
**Median age (IQR)**	68.02	71.07	NS	57.52	57.92	NS
	(57.96–73.96)	(59.29–77.31)		(51.95–63.14)	(51.35–65.65)	
**Pathologic T**			*			**
T1	15(55.6%)	9(19.6%)		5(45.5%)	3	(3.7%)
T2	3(11.1%)	8(17.4%)		1(9.0%)	30	(37.0%)
T3	9(33.3%)	28(60.9%)		5(45.5%)	44	(54.3%)
T4	0(0.0%)	1(2.1%)		0(0.0%)	4	(4.9%)
**Pathologic N**			NS			NS
N0–1	24(88.9%)	37(82.2%)		9(81.8%)	73(90.1%)	
N2–3	3(11.1%)	8(17.8%)		2(18.2%)	8(9.9%)	
**Pathologic M**			NS			NS
M0	18(85.7%)	33(93.9%)		8(80.0%)	74(97.4%)	
M1	3(14.3%)	2(6.1%)		2(20.0%)	2(3.6%)	
**Survival (Median year)**	4.88	1.67	*	3.73	1.41	**
**Stromal activation score (IQR)**	− 0.2581	0.04585	****	− 0.18993	0.20188	****
	(− 0.3445 to − 0.2140)	(− 0.04016–0.17124)		(− 0.23276 to − 0.12461)	(0.06002–0.38233)	

*IQR* interquartile range.

*NS* no significance; *P < 0.05; **P < 0.01; ****P < 0.0001.

To validate the prognostic efficiency of our stromal classification model, we collected both transcriptome and clinical data of 48 ESAD patients (GSE19417) as well as 179 ESCC patients (GSE53625) as test cohorts from Gene Expression Omnibus (GEO) database. Stromal scores were acquired via our stromal classification model using ridge Cox regression method based on the 32 hub genes expression. The test patients in both test cohorts were divided into 2 groups (S1 and S2) based on their stromal scores. Survival analysis of GSE19417 dataset revealed stromal activation scores performed well in prognostic prediction in ESAD patients (S1 vs. S2: median survival year, 3.48 vs. 1.39, P = 0.018, Fig. 6D). The same result was observed in ESCC that patients in the S2 group had a significantly lower survival probability (S1 vs. S2: median survival year, 3.31 vs. 2.03, P = 0.044, Fig. 6E). In ESCC, the S2 group had higher stromal activation scores as well as a higher proportion of T stage compared with the S1 group, suggesting that higher expression of stromal genes was related to terminal T stage and unfavorable prognosis (Table 2). Moreover, the EMT markers together with vessel angiogenesis markers also were highly expressed in the S2 group (Figs. S4, S5).

**Table 2:** Clinical information for validation patients with ESCC.

Variable	S1	S2	*P*
**Gender**			NS
Male	112(83.6%)	34(75.6%)	
Female	22(16.4%)	11(24.4%)	
**Tobacco history**			NS
Yes	85(63.4%)	29(64.4%)	
No	49(36.6%)	16(35.6%)	
**Alcohol history**			NS
Yes	83(61.9%)	23(51.1%)	
No	51(38.1%)	22(48.9%)	
**Median age (IQR)**	60.00	59.00	NS
	(53.00–66.80)	(54.00–62.12)	
**Pathologic T**			**
T1	10(7.5%)	2(4.4%)	
T2	23(17.2%)	4(8.9%)	
T3	86(64.2%)	24(53.3%)	
T4	15(11.2%)	15(33.3%)	
**Pathologic N**			NS
N0–1	61(45.5%)	22(48.9%)	
N2–3	73(54.5%)	23(51.1%)	
**Median survival year**	3.31	2.03	*
**Stromal activation score (IQR)**	0.1277	0.1411	****
	(0.1218–0.1320)	(0.1399–0.1427)	

*ESCC* esophageal squamous cell carcinoma; *IQR* interquartile range.

*NS* no significance; *P < 0.05; **P < 0.01; ****P < 0.0001.

These results were consistent with the findings in the training samples based on the TCGA dataset, confirming that stromal activation was linked to EMT, angiogenesis, advanced T stage, and poor prognosis.

### The identification of markers on stromal activation

**Figure 7:**
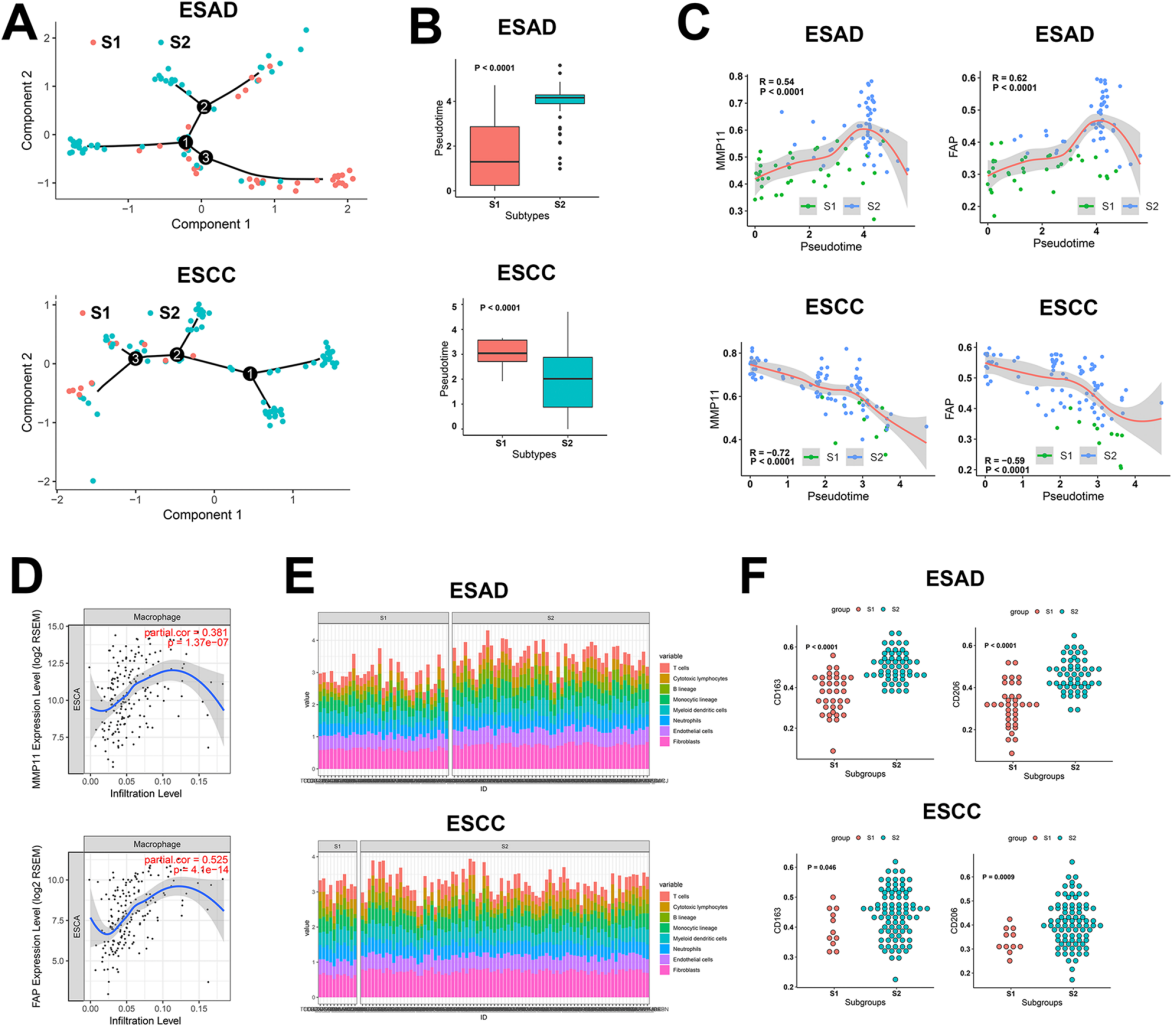
The identification of stromal activation markers and macrophage M2 polarization in stroma activated groups. **(A)** Trajectory analysis to identify stromal activation markers. **(B)** Pseudotime better reflects stromal groups. **(C)** Correlation between pseudotime and marker genes. **(D)** Marker genes linked to higher macrophage infiltration. (**E)** Cellular infiltration in stromal groups. **(F)** M2 macrophage marker expression in stromal groups.

To identify the markers of stromal activation and investigate the function of dynamic expression of these markers in classification of ESCC and ESAD, we performed trajectory analysis on both types of ESCA. As expected, the S1 and S2 group assembled at different ends of the trajectory in both cancers (Fig. 7A,B), implying the heterogeneity of development in the 2 stroma groups. Combining these results with survival analysis and correlation analysis between stroma-related signatures and pseudotime in the trajectory, we finally determined several stromal activation markers, including MMP11, COL6A2, COL1A2, CTHRC1, FAP, and LUM, which were common between ESCC and ESAD (Fig. 7C, Fig. S6). The S2 group was characterized as MMP11^high^ COL6A2^high^ COL1A2^high^ CTHRC1^high^ FAP^high^ LUM^high^, all of which were involved in CAFs basic function including fibrocyte activation, fiber synthesis, or degradation of extracellular matrix.

Through further exploration, we were able to produce a thorough portrait of the expression of the above markers. MMP11, COL1A2, CTHRC1, FAP, and LUM, but not COL6A2, were found to be upregulated in ESCA compared with normal tissues (Fig. S7). All of them showed remarkedly negative correlation with prognosis (Fig. S7), consistent with the above results.

### Immune cell infiltration profiles in different stromal subtypes: the stromal activation was linked to macrophage M2 polarization

We detected the infiltration of immune cells in tumor tissues by transcriptomics. Surprisingly, this showed that all the 6 markers had a positive correlation with macrophage infiltration (Fig. 7D, Fig. S8). Therefore, we comprehensively analyzed the cell infiltration patterns of ESAD and ESCC in different stromal subgroups (Fig. 7E). The S2 group showed more abundant cell infiltration, especially that of fibroblasts, endothelial cells, and monocyte macrophages (Fig. S9) in ESAD and ESCC. TAMs, especially M2 type, promote the growth and metastasis of tumor cells in multiple ways. To study the infiltration of TAMs in ESAD and ESCC, we utilized marker molecules expressed on the surface of macrophages to differentiate their polarization status (M1: CD83; M2: CD163, CD206). Although both M1 and M2 characteristic molecules were all highly expressed in the S2 group (Fig. 6f), M2 markers were upregulated compared with the S1 group after correcting the abundance of monocyte macrophages in ESAD and ESCC (Fig. S10). This analysis indicated that macrophages were polarizing to M2 status in ESCA patients of the S2 group. The M2 status could probably contribute to high activity in the stroma and worse survival.

Taking together, we proposed a practical classifier of the stromal microenvironment and analyzed the association between stromal activation and multi-omics characteristics. In conclusion, stromal activation was beneficial to stromal cell infiltration (fibroblasts, endothelial cells, and monocyte macrophages), EMT, angiogenesis, and M2 macrophages polarization, which were linked to poor prognosis.

## Discussion

In this study, we focused on the comprehensive stromal characterizations of ESCA cohorts based on stroma-related gene expression. A stromal activation classifier was established to quantify stromal activation of ESCA patients based on 32 stromal-related genes expression profiles using cox ridge regression. Consequently, correlation analyses between stromal activation and clinical features revealed that overactivated stroma was highly associated with higher T stage and unfavorable prognosis. A thorough portrait of stromal landscapes indicated that CAFs activation, EMT, tumor angiogenesis and M2 macrophage polarization were implicated in ESCA activated stroma, contributing to poor survival of patients. Moreover, further subgroup analysis exhibited a significant value of our stromal activation classifier on prognosis and infiltration prediction in both ESAD and ESCC, implying the crucial role of stromal activation in ESCA biological behaviors. Taken together, our findings outlined the specific biologic features of activated stroma and provided a practical stromal activation classifier with prognostic value for ESCA.

Research has shown that tumor cells rely on a detrimental microenvironment which is composed of abnormal tumor vessels, lymphatics, and tumor stroma, to develop, proliferate, invade, and transfer^13,14^. Well received ‘seed-and-soil’ hypothesis also highlighted the interaction between tumor and stroma in tumor microenvironment^15^. Stroma turns fibrotic and is activated to form a more ridge and tensor ECM during tumor development. CAFs undergo overt changes in expression levels of chemokine, cytokines and growth factors, which aggravate vast alteration of ECM and induce cancer infiltration, metastasis and recurrence^16^. The tumor stroma can restrict the delivery of drugs and induce resistance to targeted therapies, hormone antagonists, and immunotherapy, protecting tumor cells from being destroyed^5^. High tumor stromal signatures are always associated with poor prognosis^17^.

Accumulating evidence shows that TNM stage is crucial to clinical diagnosis and selection of combined therapy in ESCA patients^18^. Surgery, radiotherapy, and chemotherapy are used, independently or in combination, to treat individual ESCA patients according to their diverse TNM stages^18–20^. In clinical practice, terminal-stage patients with high levels of infiltration and metastasis need more intensive monitoring and aggressive treatment, and robust biomarkers are urgently needed to distinguish high-risk patients to improve their survival^21^. In our study, we focused on the mutual influence between the stromal microenvironment and tumor stage (infiltration) to provide a practical stromal activation classifier.

The outcome of WGCNA suggested the potentially negative correlation between T and M stages in blue and turquoise modules. Further downstream analyses revealed that it was actually T stage that was tightly related to stromal scores and OS of ESCA patients, and that the small proportion of distant metastasis patients in TCGA ESCA cohort probably led to the non-significance of M stage in statistics.

Importantly, stromal-associated signatures containing FAP, COL1A2, COL6A2, MMP11, LUM, and CTHRC1 were identified. All these signatures were involved in CAF basic function including fibrocyte activation, fiber synthesis, or degradation of extracellular matrix^22–26^. Previous researches reported that stromal relevant genes such as angioetin-1, TGF-β, connective tissue growth factor, and MMPs distinctly influenced the composition of the cancer microenvironment and the interaction with the extracellular matrix^27–30^, leading to differential T stages. In line with this, our novel identified stroma markers highlighted the essential roles of CAFs in tumor stroma. Moreover, cellular infiltration and survival analysis in our study demonstrated that patients in high activation stroma group showed high infiltration levels of CAFs, endothelial cells and M2 macrophages as well as poor prognosis. In parallel, CAFs was reported to be strongly implicated in cancer angiogenesis, metastasis and prognosis in ESCA^31^. For example, CAFs in stroma constantly secret cytokines including FAP to remodel the extracellular matrix and modulate immune cell trafficking in mouse models of breast and colon cancers^32,33^. CAFs-derived TFG-β and VEGFs relatively facilitated EMT and tumor angiogenesis in cancer stroma^6,8^, confirming our identified stromal characterizations of stroma-activated ESCA patients. Similarly, excessive vascular and lymphatic endothelial cells not only provide tumor cells with sufficient nutrition to develop and means to transfer, they have also been shown to suppress the activation of T lymphocytes and polarization of Th1 lymphocytes^34^. Moreover, TAMs, as the most cellular component in tumor stroma, were reported to induce CAF differentiation and augment tumor growth via CCL18 secretion^35^. Furthermore, CAFs were indicated to enhance TAM recruitment in tumor microenvironment by CXCL12-CXCR4 and CCL5-CCR5 axis, forming a positive feedback loop between CAFs and TAMs as well as tumor-promoting stroma^7,36^. Regarding the relevance of CAFs in survival, clinical retrospective researches revealed the CAF infiltration was negatively relevant to OS of ESCC and ESAD patients (ESCC: 16 vs. 51 months; ESAD: 46.80 vs. 76.45 months)^37,38^, which were consistent with our prediction outcome of ESCA patients based on stromal scores. The interactions of these tumor-associated cells, especially CAFs in the microenvironment provide a solid foundation for the high stromal activation observed in the S2 group.

The genomic landscape was further explored for the S1 and S2 groups. Notably, patients in the S1 group tended to bear a greater mutation burden. Meanwhile, patients in the S1 group showed mutations especially focusing on PEG3, TUBA3C, and COL12A. These genes were associated with the alteration of cell proliferation, cytoskeleton, and collagen fiber formation^39^. Hence, the stably high expression of stromal-related genes in the S2 group promoted stromal activation compared with the S1 group.

There were some limitations of this study. It had a small sample capacity, and a support from prospective study was required. In addition, it was confined to ESCA and did not consider the pan cancer cases.

In summary, a practicable classifier based on the stromal microenvironment and a quantitative index of stromal activation were proposed in this study. The relationship between cancer-promoting stromal activation and patient characteristics, including molecular features, cell infiltration, and clinical traits, were explored extensively. This study provided a novel prospective on multi-omics in patients and the tumor stromal microenvironment.

## Methods

### Data preprocessing and standardization

Three data sets consisting of RNA sequencing (RNA-Seq), gene chip, and clinical data from 409 ESCA samples were acquired from TCGA and GEO databases (TCGA ESCA, n = 182; GSE19417, n = 48; GSE53625, n = 179). Genes without expression in most samples (> 50%) were excluded from further analysis. Log_2_(x + 1) standardization and RNA-Seq by expectation maximization^40^ were applied to the RNA-Seq data. Robust multi-array average standardization was used to process the RNA profiling chip^41^. Z-score normalization was applied to all gene expression data.

### Weighted co-expression network analysis

Weighted co-expression network analysis (WGCNA)^42^ was used to identify gene expression patterns in the RNA-Seq data. We performed whole-genome hierarchical clustering and discarded outlying samples (cut-off = 90, Additional file 1: Fig. S11a,b). Genes with similar expression patterns were defined as gene modules through K-means clustering. Eigenvalues of gene modules were defined as first principal components by principal component analysis. Spearman, Pearson correlation^43^, and univariate Cox regression were performed for the order, numerical, and survival data of different phenotypes, respectively.

To construct the co-expression network, the co-expression similarity ${S}_{i,j}$ of gene I and gen j, defined as the absolute value of the Pearson coefficient, was calculated as follows:

$$S_{i,j} = \left| {{\text{per}}\;\left( {x_{i} ,x_{j} } \right)} \right|$$

An adjacency matrix was used to determine whether gene i and gene j shared the same expression module. The soft threshold was increased to make the matrix elements continuous in order to avoid rigid division.

$$a_{i,j} = {\text{power}}\;\left( {s_{i,j} } \right) = \left| {\left( {s_{i,j} } \right)} \right|^{\beta 1}$$

The parameter of the power function (β) was selected to provide a signed scale-free co-expression gene network as the soft-thresholding parameter (power = 4, Additional file 1: Fig. S12). As the similarity between two genes is not determined only by their expression similarity, a topological overlap matrix was constructed to explore the indirect relationships between gene i and gene j via an intervening gene u.

$${\text{W}}_{ij} = \frac{{l_{ij} + a_{ij} }}{{\min \left\{ {k_{i} ,k_{j} + 1 - a_{ij} } \right\}}}\;\;{\text{where}}\;l_{ij} = \mathop \sum \limits_{u} a_{iu} a_{uj} ,\;k_{i} = \mathop \sum \limits_{u} a_{iu}$$

### Random forest regression

RF regression^44^ was applied to train labeled data and to predict unlabeled data by constructing multiple decision trees and merging them for more accurate and stable prediction. Mean square error was quantified by the expected value of the square of the difference between the estimated value and the true value of the parameter (Additional file 1: Fig. S13a–d). A random forest regression model was used to screen genes using mean square error and node purity^45^.

### Protein–protein interaction and network analysis

We explored interacting proteins with similar function in material metabolism, cell cycle regulation, biological signal transduction, gene expression regulation, energy, and other aspects of life processes based on miscellaneous evidence (gene fusions, gene co-occurrence, text mining, co-expression, and protein homology) from the String database (https://string-db.org/)^46^. In the PPI network, topological structure was estimated using betweenness, closeness, and degree, where degree was defined as the number of genes interacting with a certain gene, betweenness refers to the frequency with which a gene acts as the shortest pathway between another two genes, and closeness means the average length of the shortest interaction pathway with other genes.

### Ridge Cox regression

Ridge Cox regression is a biased estimation regression algorithm targeted at colinear data sets. Ridge regression uses L2 regularization^47,48^ to avoid overfitting and promote generalization, exhibiting less sensitivity to extreme variation (such as outliers). Compared with the ordinary Cox regression model, ridge regression exhibits more generality:

$${\text{L}} = E_{in} + \lambda \sum iW_{i}$$

where ${E}_{in}$ is the training sample error without a regularization term in the Cox model, $\uplambda$ is the L2 regularization coefficient, and ${w}_{i}$ is the weight of the gene i.

Stromal-activated genes were selected via the receiver operating characteristic curve, PPI network, RF regression, and hierarchical clustering. The ridge regression algorithm was then used to construct a stromal-based prognostic model with ridge Cox regression using the glmnet package (Additional file 1: Fig. S14a,b).

### Gene set variation analysis

Gene set variation analysis for microarray and RNA-Seq data^49^ was used to appraise variations in pathway activity over every single sample in an unsupervised way. Gene rank lists were sorted according to gene expression levels in a single sample. The samples with top- or bottom-ranking genes were considered to be highly enriched in in specific pathways.

### Differential gene expression analysis

DGE analysis was performed to identify genes with different expression levels in corresponding groups^50^. Relative analysis was achieved with the limma package in the R language.

### Decision curve analysis

DCA is an algorithm to estimate whether a model is worth using in clinical medicine, or which of several alternative models should be utilized, based on patients’ benefit^51^. A loss function is introduced into the regression analysis to calculate net benefit based on the benefit and loss after deciding whether to use a clinical intervention. The Rmda package was applied to achieve DCA in the R language.

### Gene enrichment analysis

Gene ontology (GO) enrichment analysis^52^ was used to identify the molecular function, biological process, and cellular component of genes (https://www.geneontology.org). Gene set enrichment analysis (GSEA) is an enrichment method used to define the molecular function of different phenotypes^53,54^. A permutation test was used to define whether the bottom- or top-ranked genes were enriched in pre-ordered gene sets. The above enrichment methods were performed by R ClusterProfiler packages, the webgestalt tool (https://www.webgestalt.org/option.php), and GSEA software.

### Survival analysis

Kaplan–Meier survival estimation^55^, a non-parametric approach to acquire survival probability by inspecting survival time, was implemented in R 3.5.1 (https://www.r-project.org/), using the survival and survminer packages to compare the prognoses of different groups. For quantitative index analysis, Cox proportional hazards regression was performed with the R survival package.

### Statistical analysis

All statistical analysis was performed using R 3.5.1. Spearman and Pearson correlation were calculated with the stats package. ROC and area under curve (AUC) calculations^56,57^ used the pROC packages. The Benjamini and Hochberg method^58^ was used to adjust the P-values of multiple testing in the enrichment and DGE analyses. P-values less than 0.05 were considered statistically significant in all hypothesis tests. All the P-values were two-sided.

## Supplementary information

Supplementary Figures.

Supplementary Tables.

## Data Availability

The datasets generated and/or analyzed during the current study are available in the TCGA (https://cancergenome.nih.gov) and GEO (https://www.ncbi.nlm.nih.gov/geo/) repository.

## Abbreviations

ESCA: Esophageal carcinoma
ESCC: Esophagus squamous cell carcinoma
ESAD: Esophagus adenocarcinoma
ECM: Extracellular matrix
CAFs: Carcinoma-associated-fibroblasts
MMP: Matrix metalloproteinase
TGF-β: Transform growth factor-β
EMT: Epithelial-mesenchymal transition
TAMs: Tumor-associated macrophages
VEGF: Vascular endothelial growth factor
TCGA: The Cancer Genome Atlas
GEO: Gene Expression Omnibus
WGCNA: Weighted correlation network analysis
RF: Random Forest
PPI: Protein–protein interaction
ROC: Receiver operating characteristic curve
DCA: Decision curve analysis
DGE: Differential gene expression
GO: Gene ontology
GSEA: Gene set enrichment analysis
AUC: Area under curve
OS: Overall survival
FDR: False discovery rate
COL6A2: Collagen Type VI Alpha 2 Chain
COL8A2: Collagen Type VIII Alpha 2 Chain
COL10A1: Collagen Type X Alpha 1 Chain
TUBA3C: Tubulin alpha 3c
PEG3: Paternally expressed 3
FAP: Fibroblast activation protein
CDH2: Cadherin 2
TWIST1: Twist Family BHLH Transcription Factor 1
