# Supplementary material for: Stromal microenvironment promoted infiltration in esophageal adenocarcinoma and squamous cell carcinoma: a multi-cohort gene-based analysis
Source: Sci Rep. 2020 Oct 29;10:18589. doi: 10.1038/s41598-020-75541-4 (PMC7596515; doi:10.1038/s41598-020-75541-4)
Supplement: Supplementary file 1 — Supplementary Figures. [file 41598_2020_75541_MOESM1_ESM.docx]

**SUPPLERMENTARY MATERIAL**

**Stromal microenvironment promoted invasion in esophageal adenocarcinoma and squamous cell carcinoma: a multi-cohort gene-based analysis**

Jiali Li^1^ · Zihang Zeng^1^ · Xueping Jiang^1^ · Nannan Zhang^1^ · Yanping Gao^1^ · Yuan Luo^1^ · Wenjie Sun^1^ · Shuying Li^1^ · Jiangbo Ren^2^ · Yan Gong^2,3^ · Conghua Xie^1,4,5^

^1^Department of Radiation and Medical Oncology, Zhongnan Hospital of Wuhan University, Wuhan, China; ^2^Department of Biological Repositories, Zhongnan Hospital of Wuhan University, Wuhan, China; ^3^Human Genetics Resource Preservation Center of Hubei Province, Zhongnan Hospital of Wuhan University, Wuhan, China; ^4^Hubei Key Laboratory of Tumor Biological Behaviors, Zhongnan Hospital of Wuhan University, Wuhan, China; ^5^Hubei Cancer Clinical Study Center, Zhongnan Hospital of Wuhan University, Wuhan, China

*Correspondence to Dr. Conghua Xie, Department of Radiation and Medical Oncology, Zhongnan Hospital of Wuhan University, 169 Donghu Road, Wuhan, Hubei 430071, China. Tel: +86-27-67812607; Fax: +86-27-6781-2892; Email: [chxie_65@whu.edu.cn](mailto:chxie_65@whu.edu.cn)

Dr. Yan Gong, Department of Biological Repositories, Zhongnan Hospital of Wuhan University, 169 Donghu Road, Wuhan, Hubei 430071, China. Tel: +86-27-67811461; Fax: +86-27-67811471; Email: [yan.gong@whu.edu.cn](mailto:yan.gong@whu.edu.cn)

**Fig. S1 Enrichment analysis of the 71 stroma-related genes.**


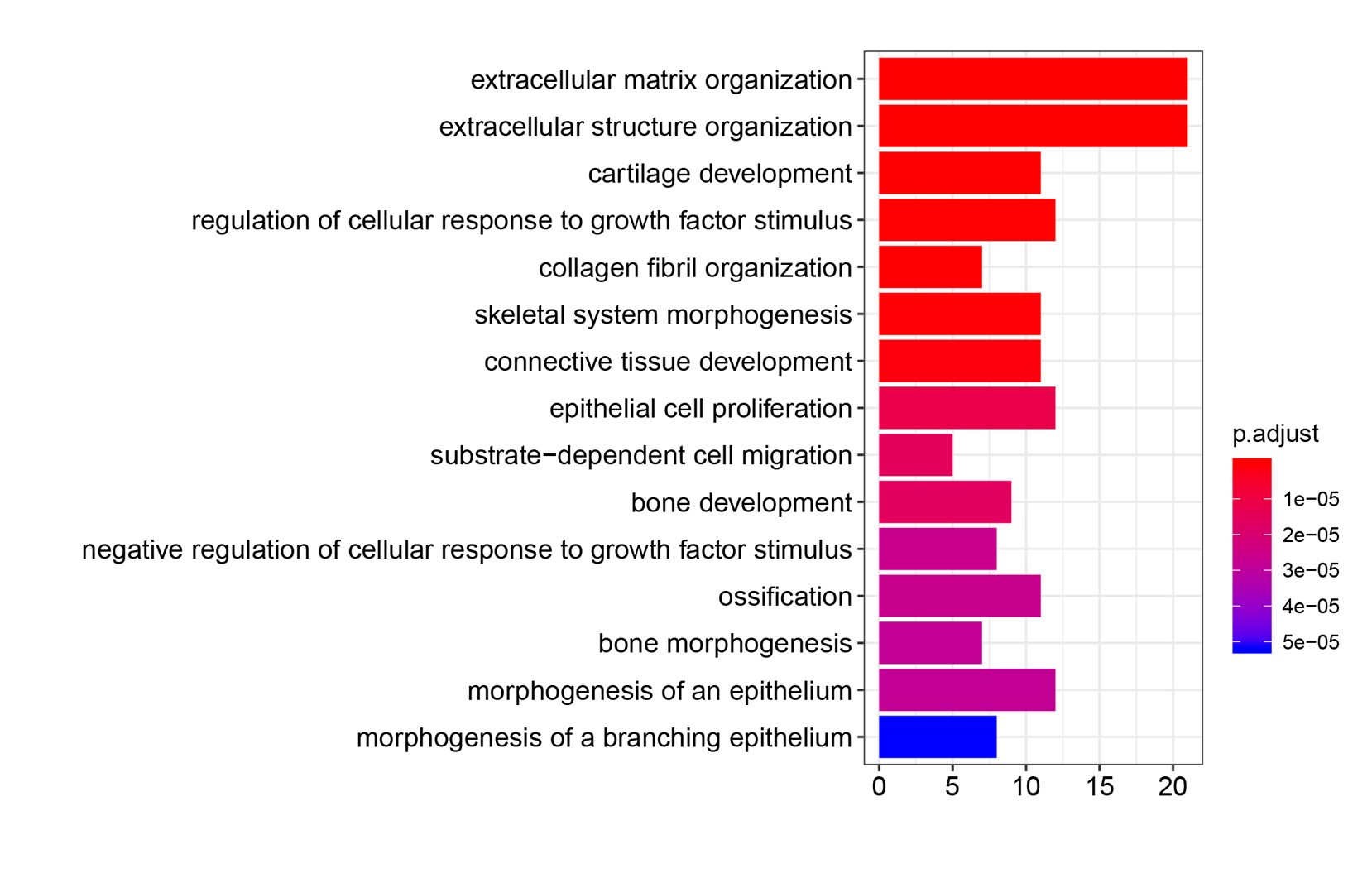


**Fig. S2 Screening for the 32 stromal characteristic genes.**

**a** Circle plot of enrichment analysis by 71 stroma-related genes. **b** Hierarchical clustering was performed to divide ESCA samples with distinct survival based on the 71 stroma-related genes. **c** Volcano map of gene expression in different stromal groups. **d** Heat map of 32 stromal characteristic genes in different stromal groups.


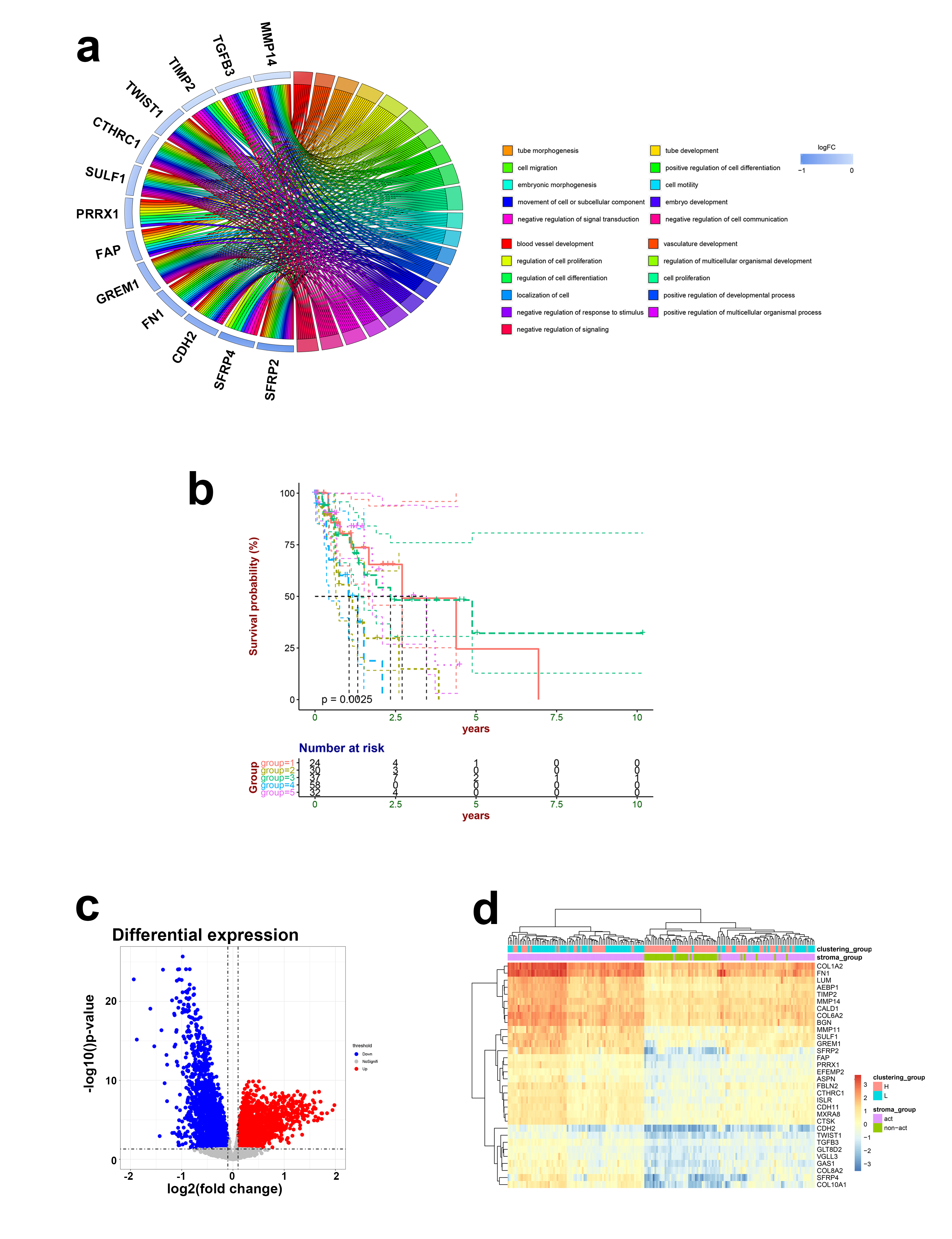


**Fig. S3 Mutation situation in different stromal groups.**

**a** Basic information of mutation in different stromal groups. **b** Tumor mutation counts of each patient in different stromal groups. **c** Mutations of different frequencies.


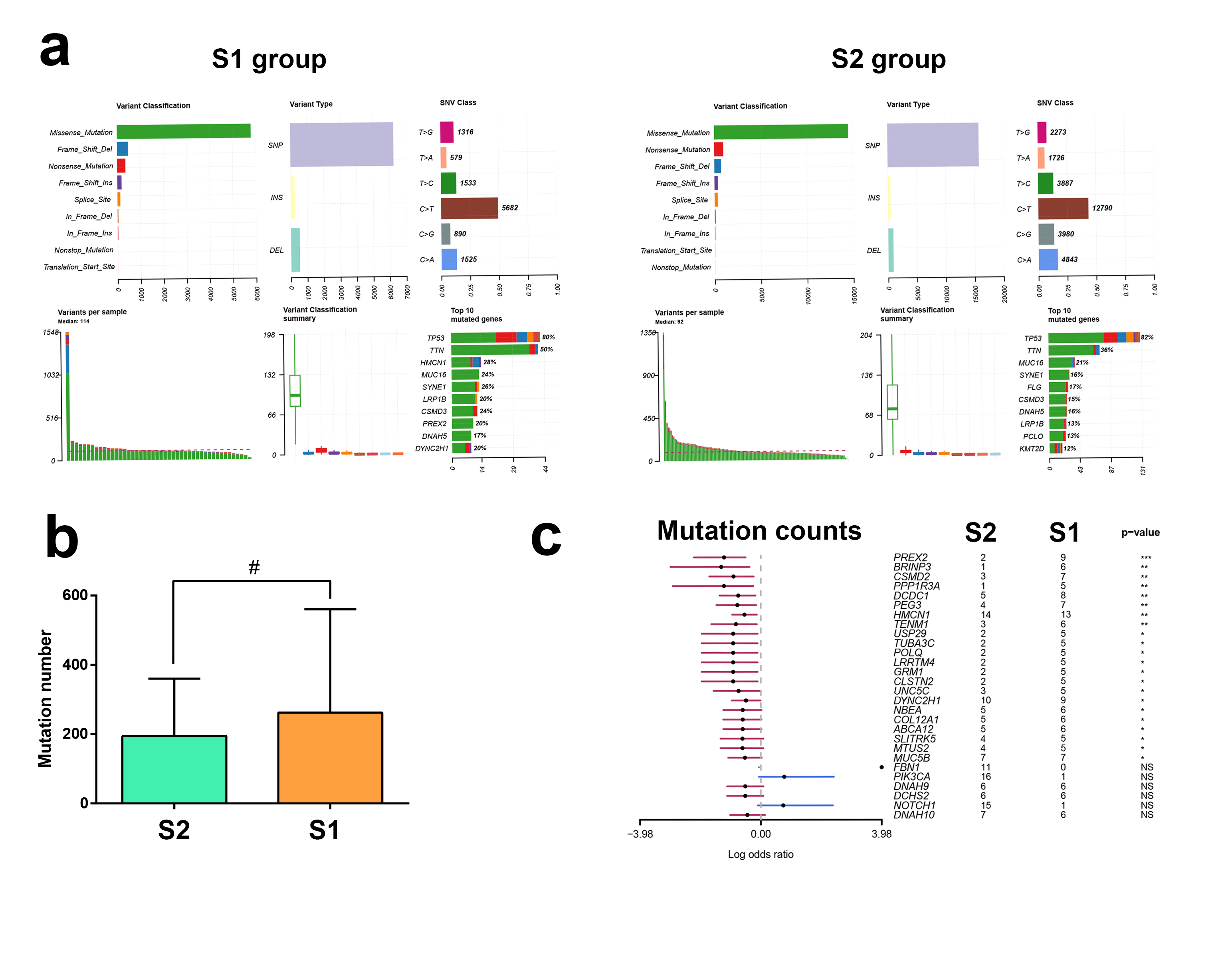


**Fig. S4 EMT markers in different stromal groups.**


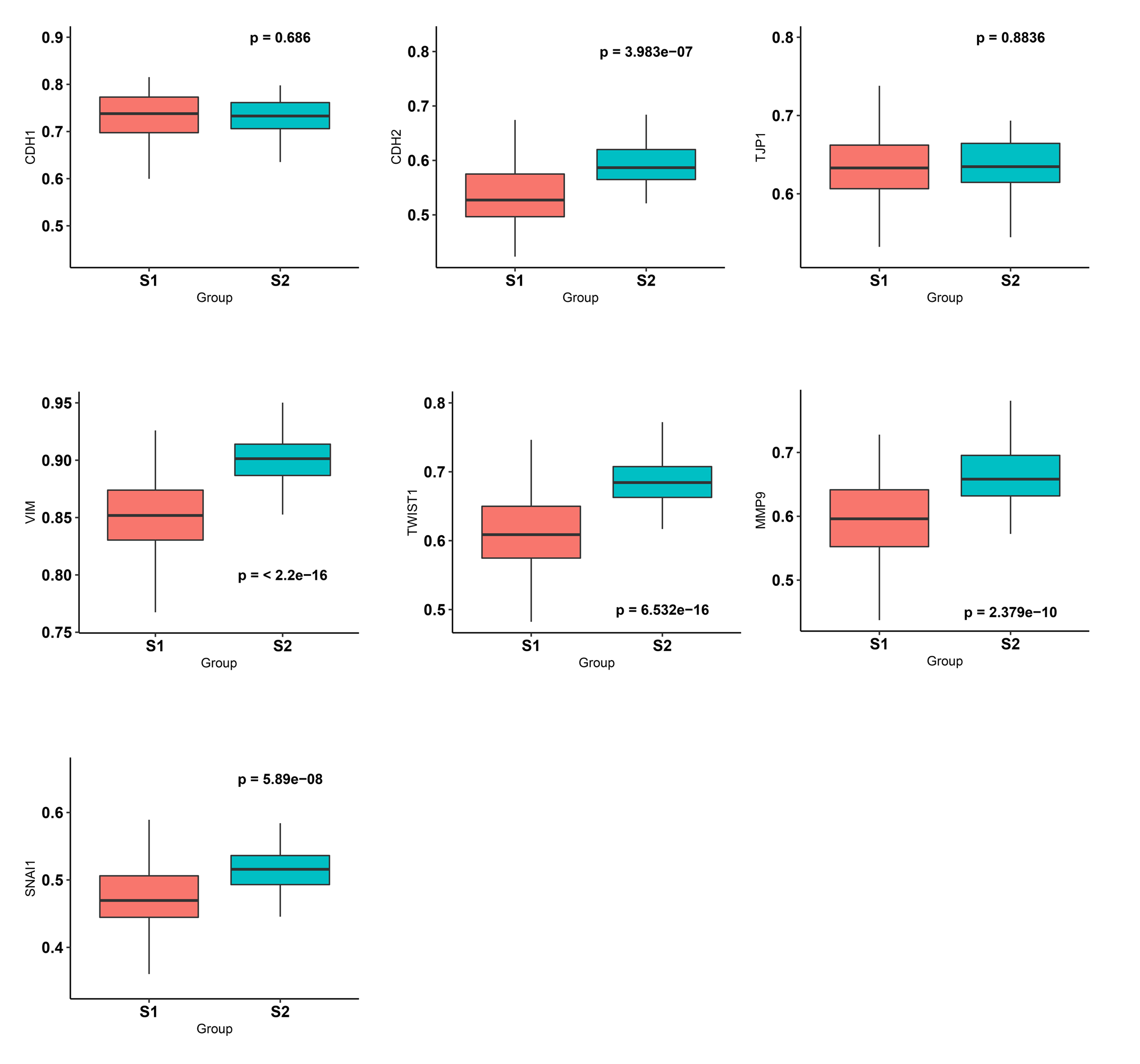


**Fig. S5 Vessel normalization markers in different stromal groups.**


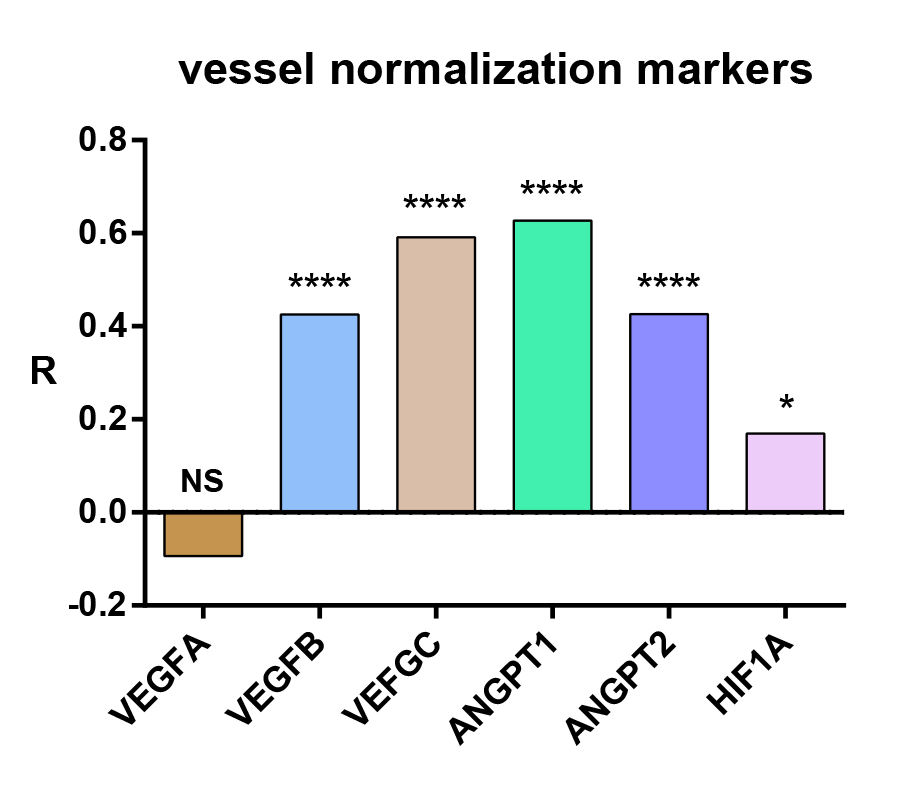


**Fig. S6 Correlation between maker gene expression and pseudotime in Trajectory analysis.**


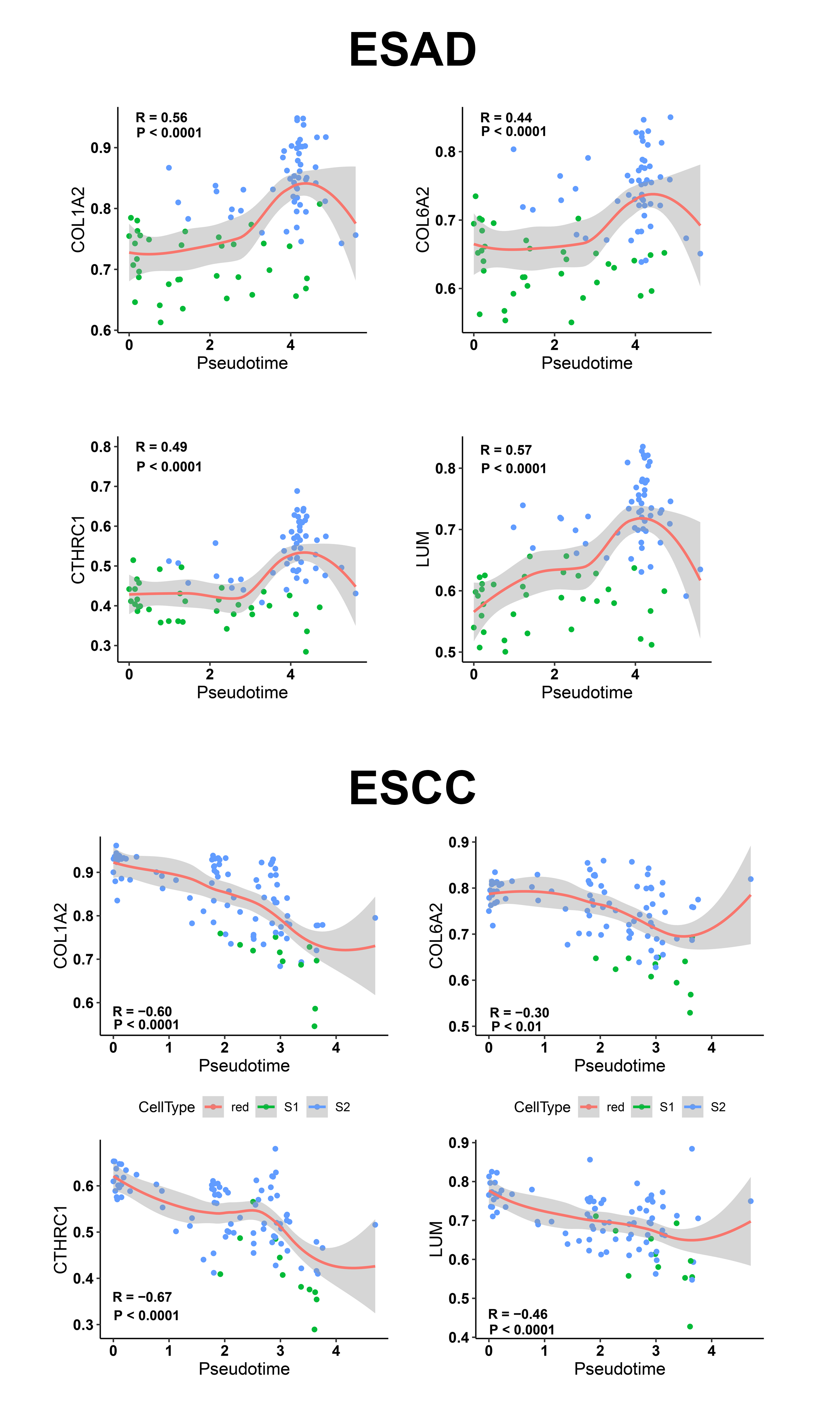


**Fig. S7 Expression of the 6 maker genes in tumor vs. normal tissues and their link to unfavorable prognosis.**


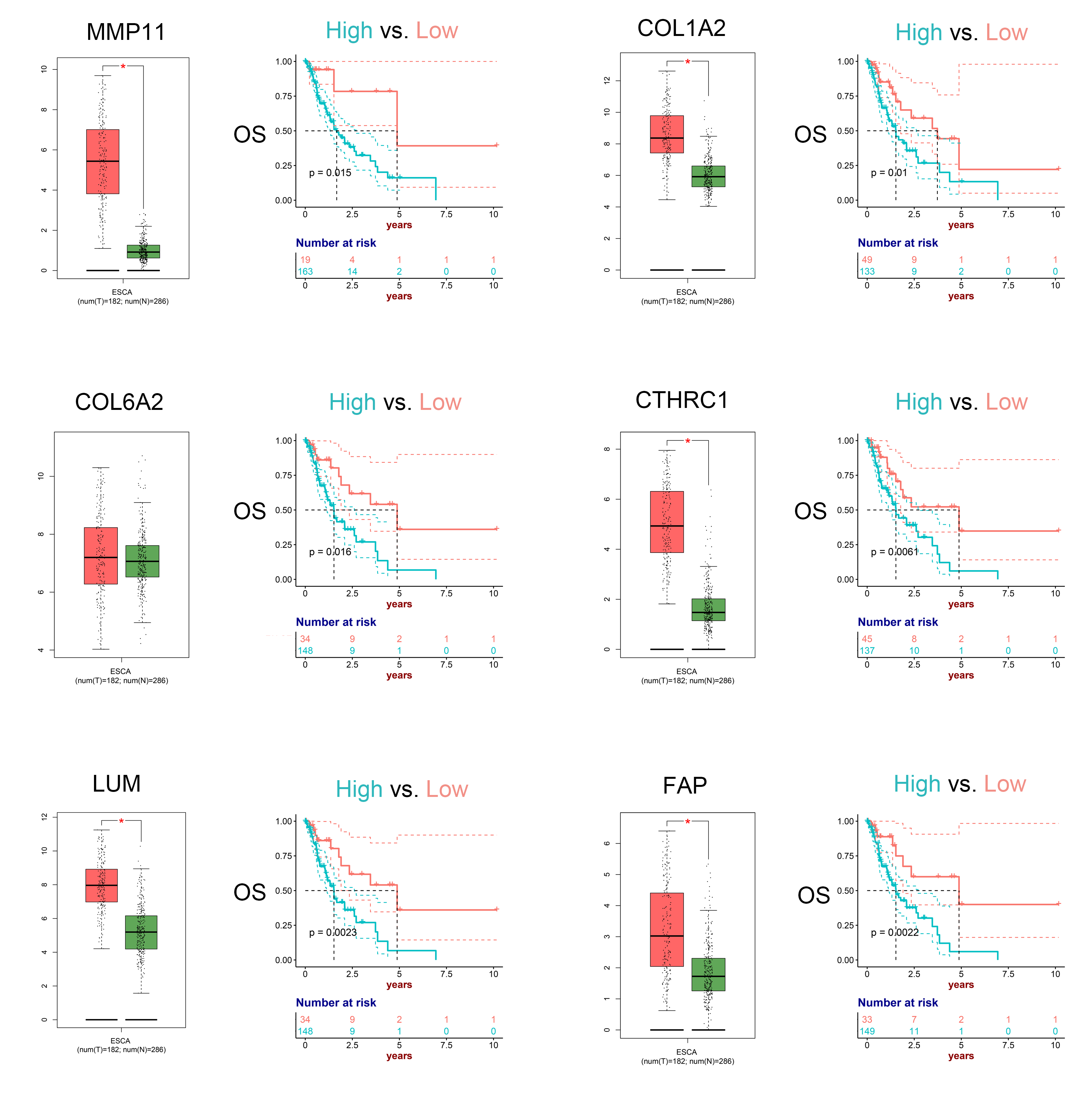


**Fig. S8 Correlation between gene expression and macrophage infiltration.**


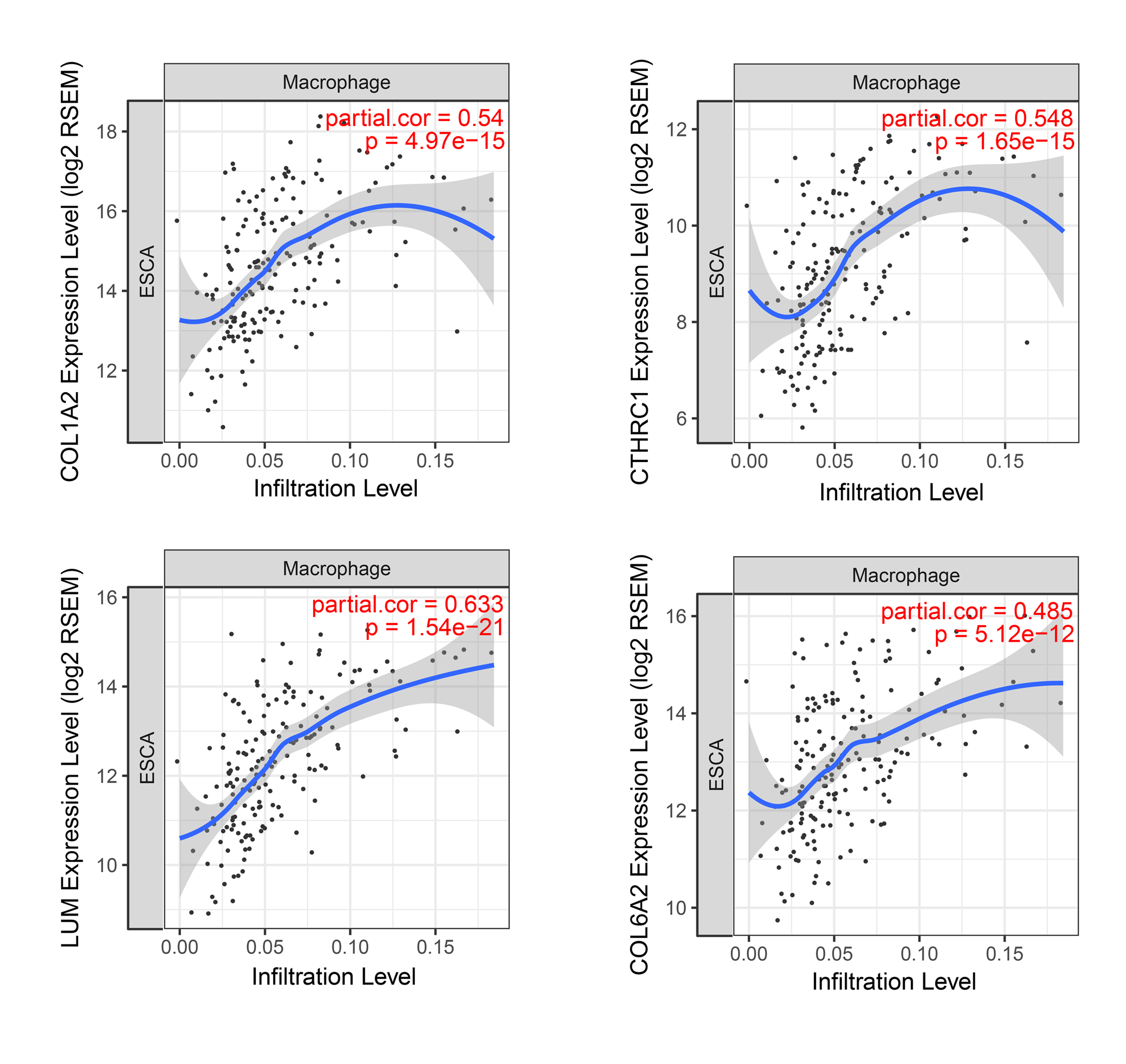


**Fig. S9 Cell infiltration in different stromal groups.**


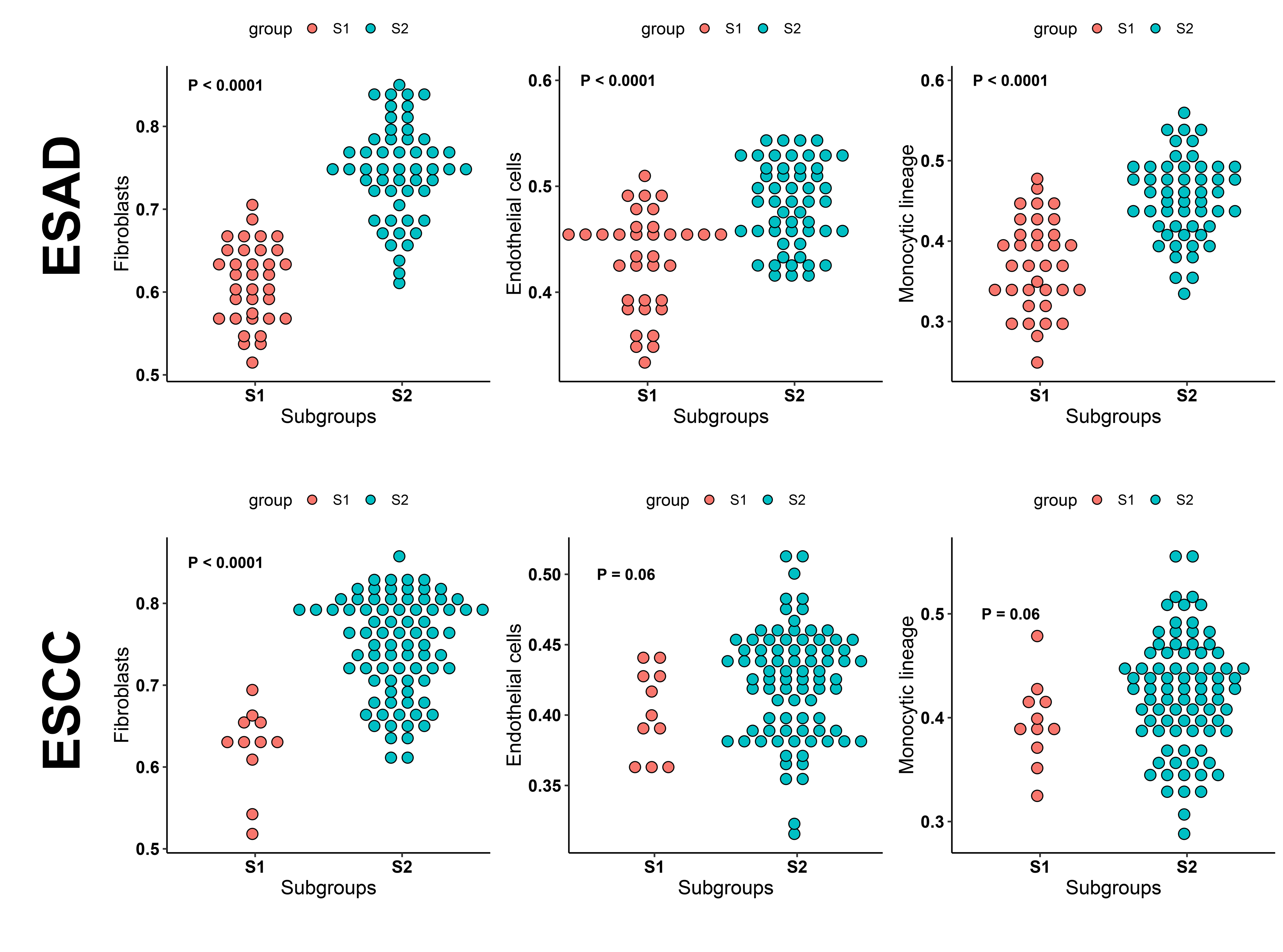


**Fig. S10 Macrophage polarization markers in different stromal groups.**


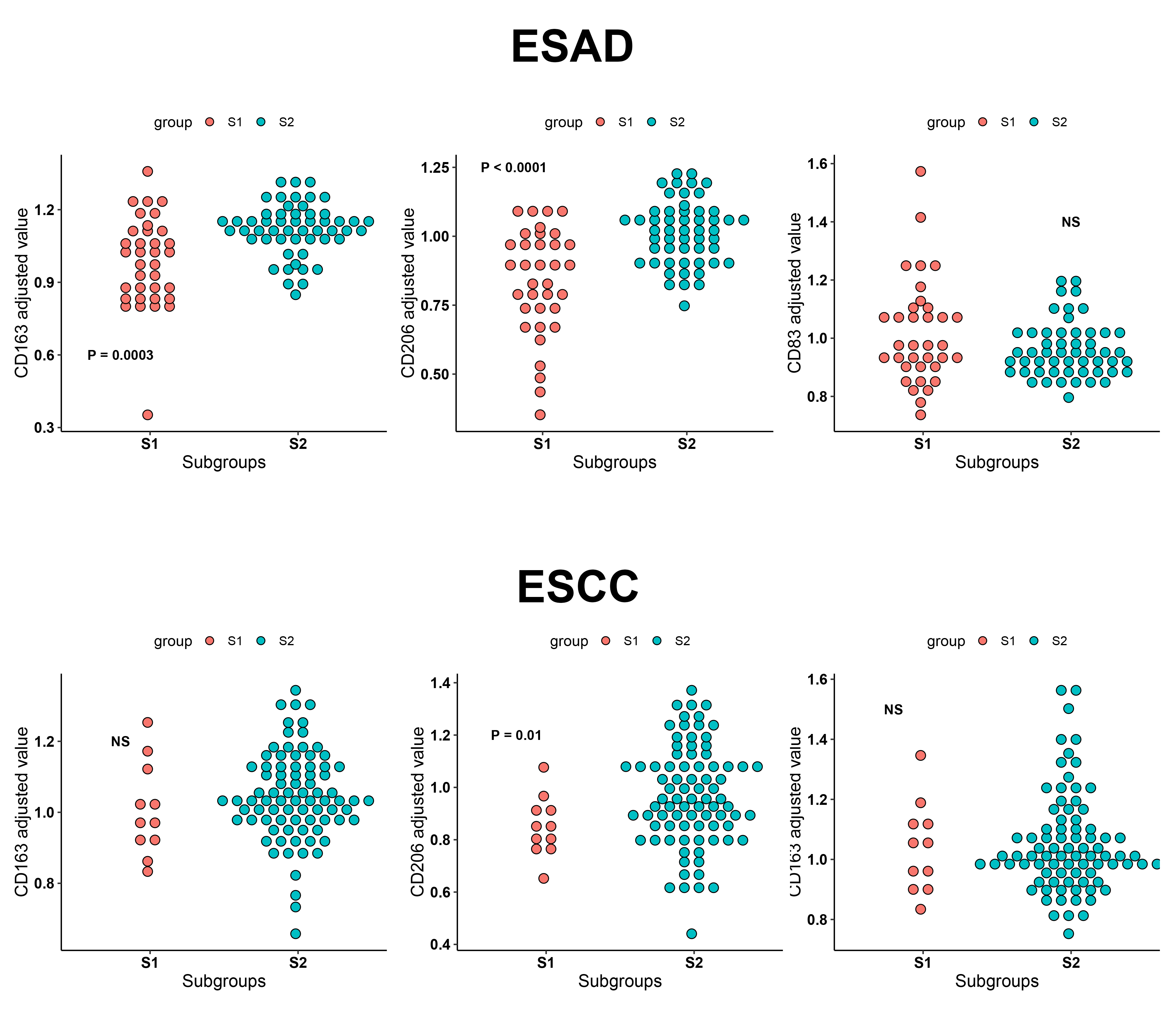


**Fig. S11 Sample clustering for the training samples in ESCA.**

**a** The number 90 is selected as threshold to exclude outlier samples. Three ESCA samples are excluded according to hierarchical clustering and 182 ESCA patients are used to furthermore analysis. **b** The 182 ESCA samples are clustered in 4 group according to hierarchical clustering based on heterogeneity.

| **a**  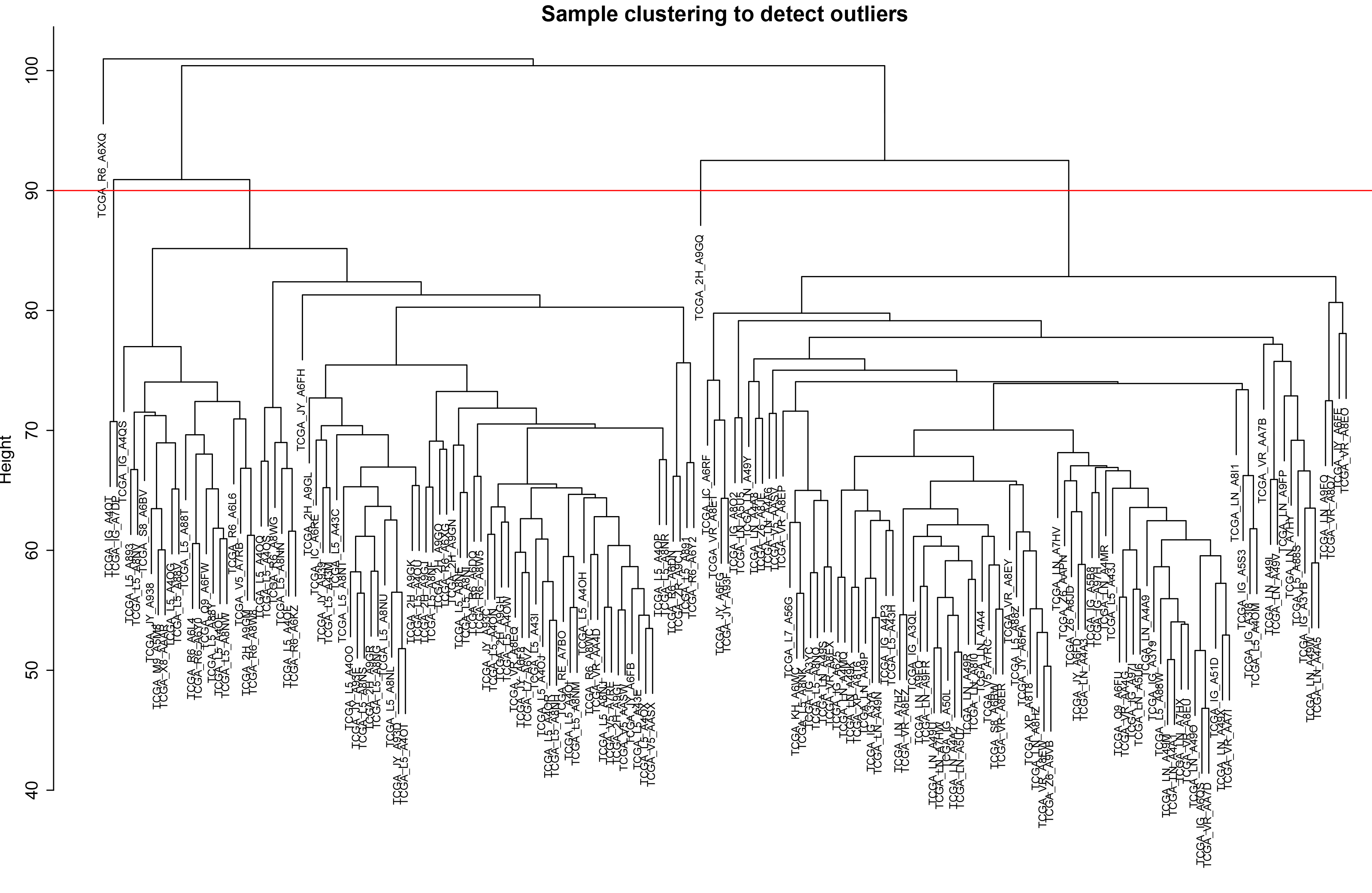 |
| --- |
| **b**  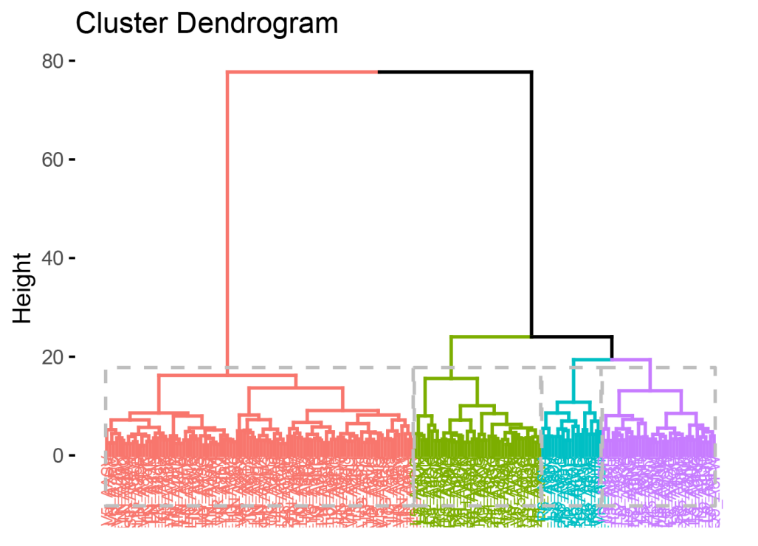 |

**Fig. S12 Scale-free co-expression gene network construction.**

**a** Power = 4 is selected as soft threshold parameter to avoid rigid gene pattern division and construct scale-free distribution co-expression gene network. **b** Mean connectivity of different parameters to test the rationality of selection for soft threshold.

| **a**  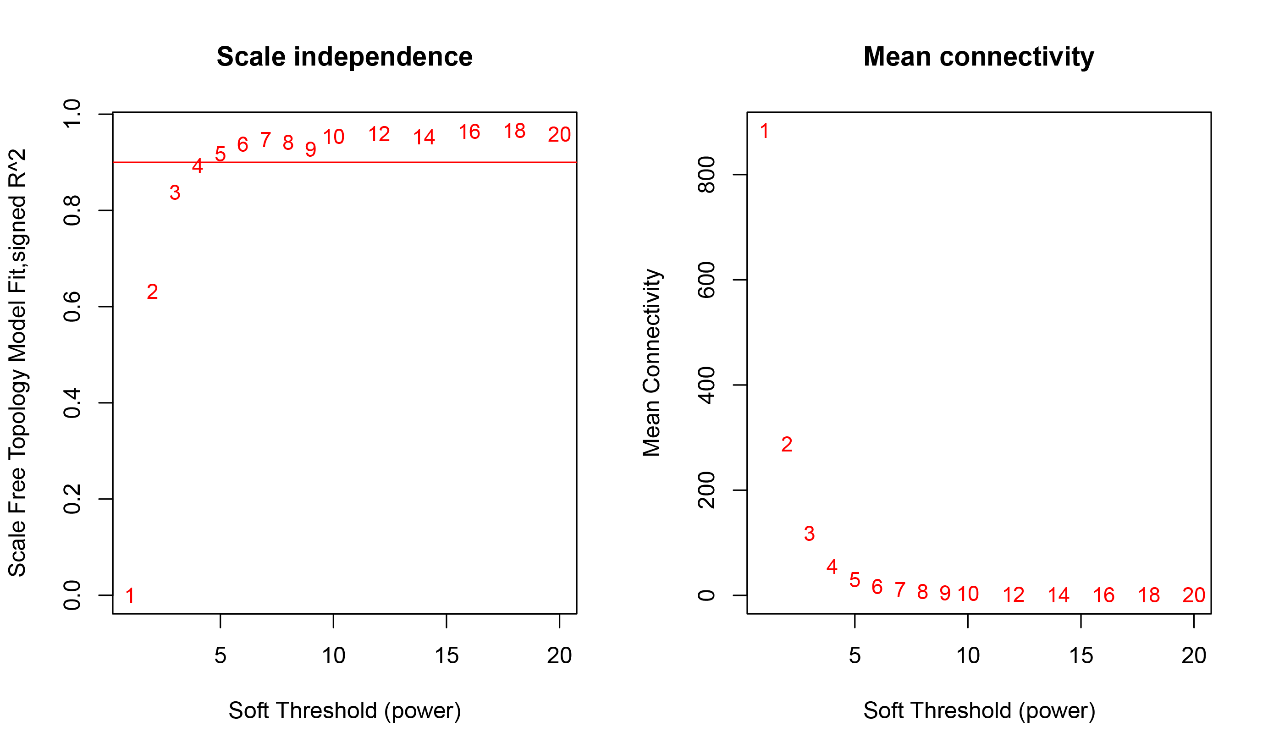 | **b** 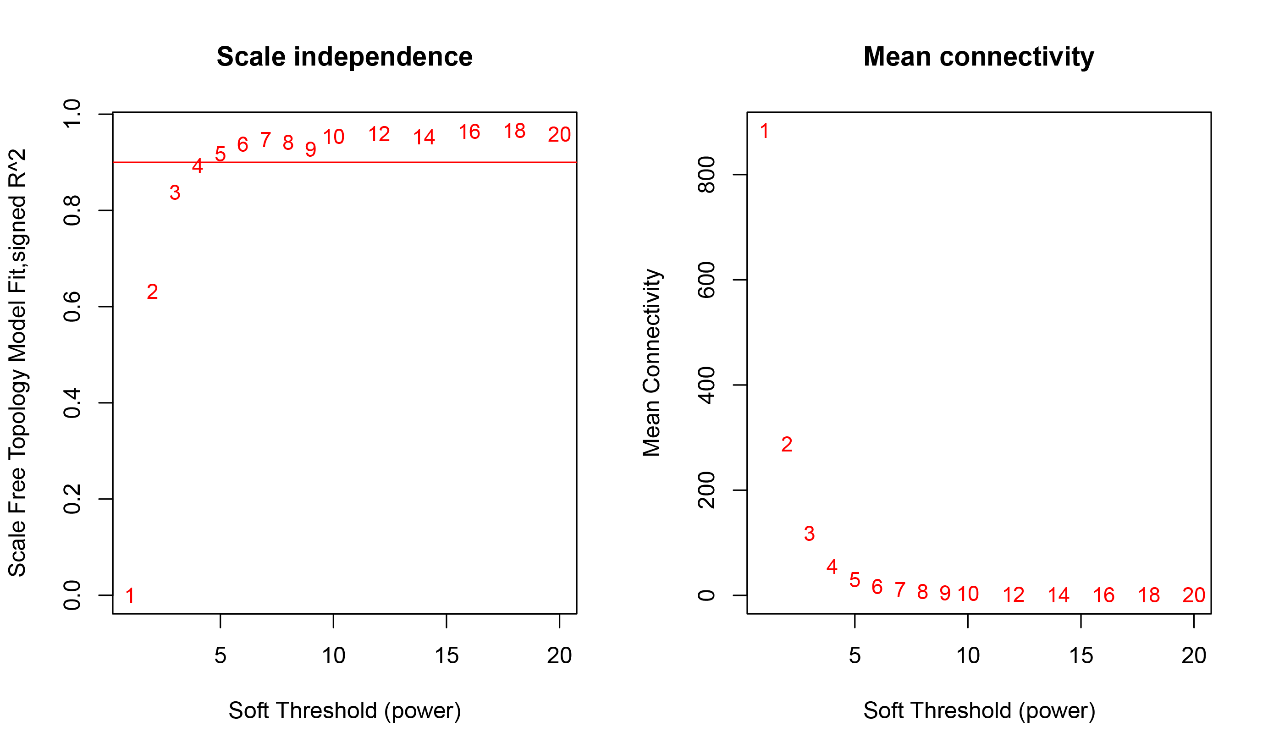 |
| --- | --- |

**Fig. S13 Index selection of random forest in T and M stage.**

**a** Mean square error to different quantity of decision trees in T stage by random forest algorithm. The quantity index of trees are confined between 0 and 150. **b** Root Mean Squared Error to different quantity of decision trees in T stage to confirm best index. **c** Mean square error to different quantity of decision trees in M stage by random forest algorithm. The quantity index of trees is also confined between 0 to 150 based on decision efficiency and calculating time. **d** Root Mean Squared Error to different quantity of decision trees in M stage to confirm best index.

| **a**  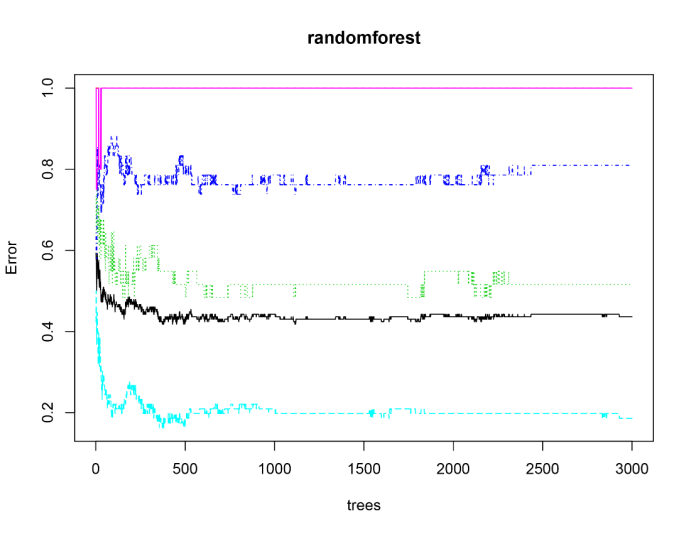 | **b**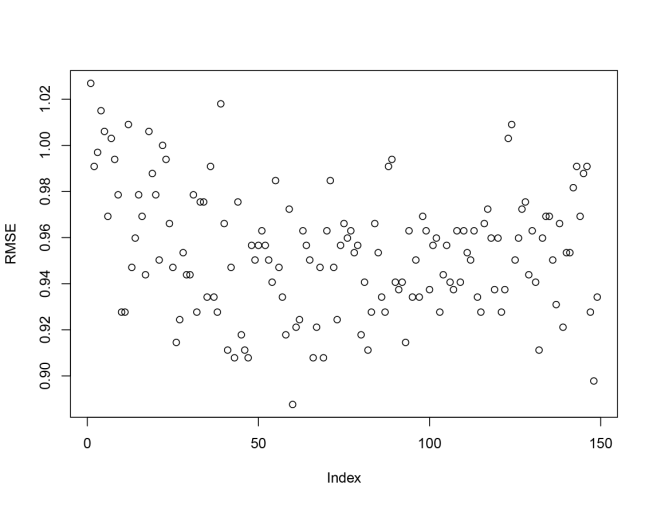 |
| --- | --- |
| 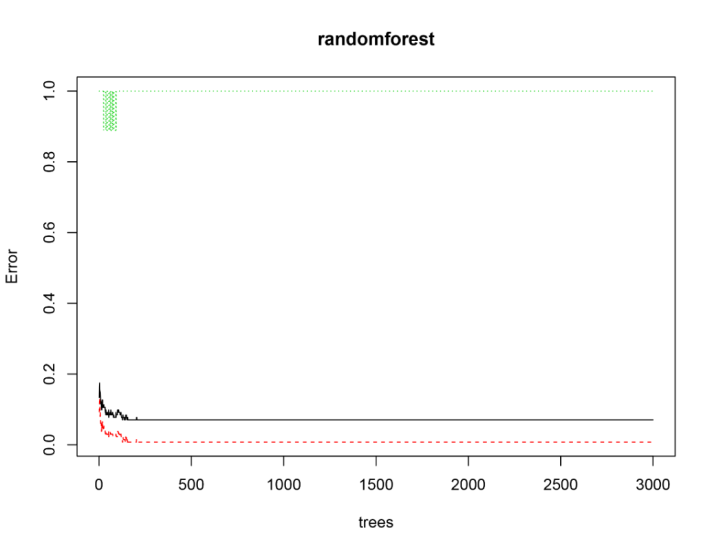**c.** | **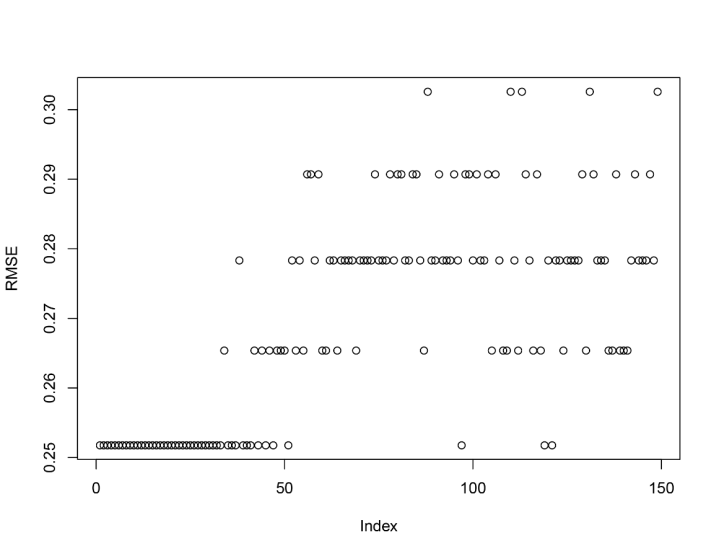d.** |

**Fig. S14 Index selection of ridge cox regression.**

**a** Selection of different regularization model. Alpha = 0 means ridge regression, alpha = 0.5 represents elastic regression and alpha =1. Log (Lambda) serves as penalty item. **b** Coefficients curve of different indexes in ridge regression constructed on L2 normalization.

| 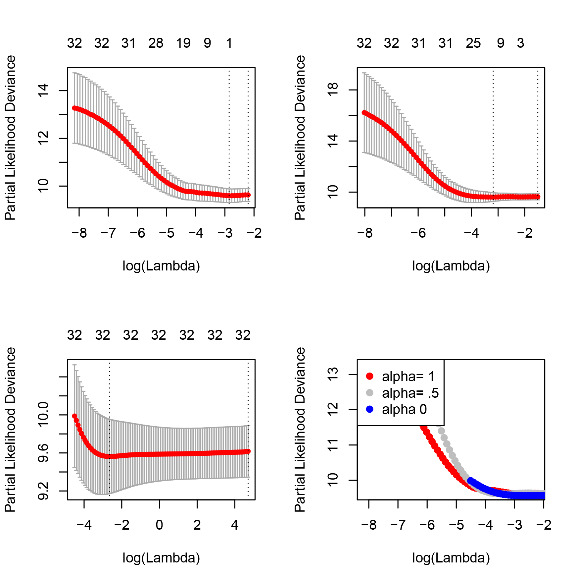a. | 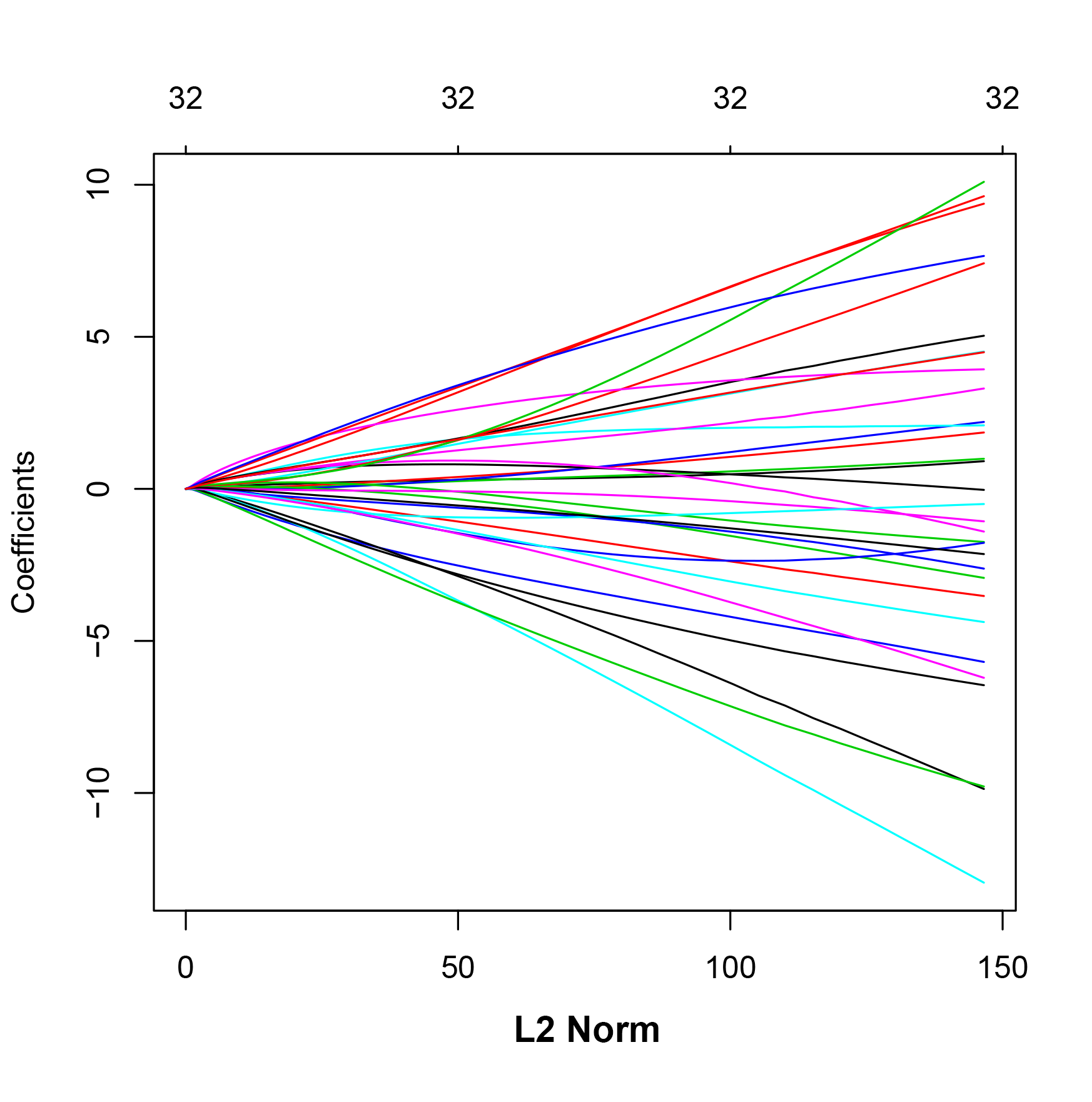b. |
| --- | --- |
